# Comparative analysis of cancer cell responses to targeted radionuclide therapy (TRT) and external beam radiotherapy (EBRT)

**DOI:** 10.1186/s13045-022-01343-y

**Published:** 2022-08-31

**Authors:** Michal Grzmil, Paul Boersema, Ashish Sharma, Alain Blanc, Stefan Imobersteg, Martin Pruschy, Paola Picotti, Roger Schibli, Martin Behe

**Affiliations:** 1 Center for Radiopharmaceutical Sciences, Paul Scherrer Institute, Villigen, Switzerland; 2 Institute of Molecular Systems Biology, Department of Biology, ETH Zurich, Zurich, Switzerland; 3 Department of Radiation Oncology, University Hospital Zurich, Zurich, Switzerland; 4 Department of Chemistry and Applied Biosciences, ETH Zurich, Zurich, Switzerland

**Keywords:** CCKBR, Minigastrin, Phosphoproteomics, Radioresistance, Erlotinib

## Abstract

The vast majority of our knowledge regarding cancer radiobiology and the activation of radioresistance mechanisms emerged from studies using external beam radiation therapy (EBRT). Yet, less is known about the cancer response to internal targeted radionuclide therapy (TRT). Our comparative phosphoproteomics analyzed cellular responses to TRT with lutetium-177-labeled minigastrin analogue [^177^Lu]Lu-PP-F11N (β-emitter) and EBRT (ɣ-rays) in CCKBR-positive cancer cells. Activation of DNA damage response by p53 was induced by both types of radiotherapy, whereas TRT robustly increased activation of signaling pathways including epidermal growth factor receptor (EGFR), mitogen-activated protein kinases (MAPKs) or integrin receptor. Inhibition of EGFR or integrin signaling sensitized cancer cells to radiolabeled minigastrin. In vivo, EGFR inhibitor erlotinib increased therapeutic response to [^177^Lu]Lu-PP-F11N and median survival of A431/CCKBR-tumor bearing nude mice. In summary, our study explores a complex scenario of cancer responses to different types of irradiation and pinpoints the radiosensitizing strategy, based on the targeting survival pathways, which are activated by TRT.

**Supplementary Information:**

The online version contains supplementary material available at 10.1186/s13045-022-01343-y.

## To the editor,

**Fig. 1:**
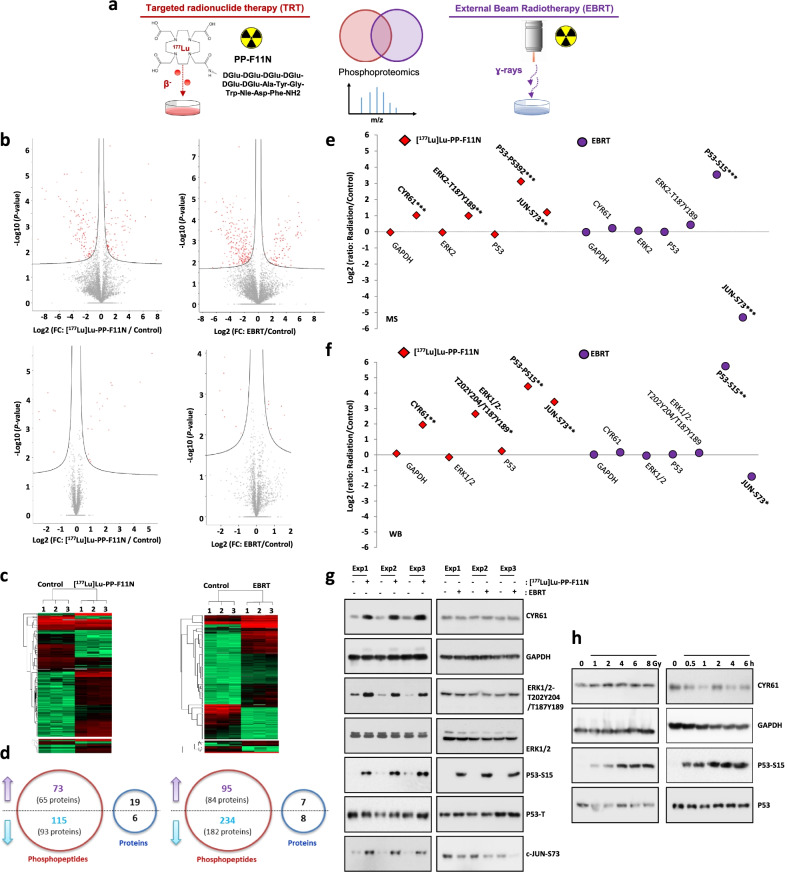
Cellular responses to TRT and EBRT. **a** A431/CCKBR cells were treated with [^177^Lu]Lu-PP-F11N or exposed to EBRT and the generated tryptic peptides and phosphopeptide enriched samples were subjected to mass spectrometry analysis. **b** Charts display phosphopeptide (upper panels) and protein (lower panels) abundance changes shown as log2 transformed fold change (FC). Red dots represent phosphopeptides or proteins with significantly altered abundance. *FDR* < 0.05. **c** Hierarchical clustering of identified changes in phosphopeptide and protein abundance. **d** Number of phosphopeptides and protein abundance changes. Arrows indicate up-regulated (purple) or down-regulated (blue) phosphorylation or protein levels. **e** MS2-based quantification of abundance changes was shown as Log2 transformed radiation/control ratios for CYR61, GAPDH, ERK2 and P53 protein levels and phosphorylation of ERK, P53 and JUN after exposure to [^177^Lu]Lu-PP-F11N and EBRT in A431/CCKBR cells. **f**, **g** Expression and phosphorylation levels of the same proteins were validated by quantitative WB analysis on total protein lysates isolated from untreated (control) and [^177^Lu]Lu-PP-F11N- or EBRT-treated cells. Each treatment was performed in triplicate (Exp 1–3). Quantification of signal intensities (in **f**) is shown as Log2 transformed radiation/control ratios as described above. *P < 0.05, **P < 0.01, ***P < 0.001. **h** WB analysis for CYR61 and P53 phosphorylation in total protein lysates isolated from A431/CCKBR cells untreated (control) and treated with 1, 2, 4, 6 and 8 Gy at indicated time points. Blot was re-probed with antibody against GAPDH and total P53

Systemic TRT employs radiopharmaceuticals that specifically target primary tumors and metastatic lesions [1]. Yet, the cancer radioresistance limits the efficacy in clinic. In 2018, FDA-approved lutathera for the first-in-class peptide receptor radionuclide therapy (PRRT) of somatostatin receptor-positive gastroenteropancreatic and neuroendocrine tumors and, recently, pluvicto has been approved for targeted radioligand therapy of PSMA-positive, metastatic castration-resistant prostate cancers [2, 3]. These advancements bring many opportunities and challenges. To explore TRT radiobiology and identify radiosensitizing strategies, we analyzed cellular responses to radiolabeled minigastrin [^177^Lu]Lu-PP-F11N that targets overexpressed cholecystokinin B receptor (CCKBR) in human cancers, including medullary thyroid carcinoma (MTC), gliomas, small cell lung and ovarian cancer [4], in comparison to EBRT (Fig. 1a). In both treatments, selected radiation doses had similar effect on cell proliferation (Additional file 1: Fig. S1). Phosphoproteomics quantified abundance of 6173 and 7293 phosphopeptides, whereas corresponding proteomics quantified 2567 and 2582 protein groups in response to TRT and EBRT, respectively (Fig. 1b). Abundance of 188 and 329 unique phosphopeptides (Fig. 1c, Additional file 2: Table S1–S4) and 25 and 15 proteins (Fig. 1c, Additional file 2: Tables S5 and S6) was significantly changed in response to TRT and EBRT, respectively (Fig. 1d). Of these, the phosphorylation of 34 proteins was common to both types of radiation. Bioinformatics analysis identified interaction networks (Additional file 1: Fig. S2) and over-represented terms for gene ontology and signaling pathways among proteins with altered level or phosphorylation in response to TRT and EBRT. Both radiotherapies influenced responses to DNA damage, signal transduction by p53, cell cycle regulation, RNA processing and metabolism as well as cellular transport, morphology and adhesion (Additional file 2: Table S7). TRT influenced DNA repair via translesion synthesis (TLS), whereas EBRT induced both double-strand break repair via non-homologous end joining (NHEJ) and homologous recombination (HR) as well as base-excision repair (BER) and interstrand cross-link repair (ICLR). Signaling of TGFβR, EGFR, HGFR, mTOR, MAPK, RAS homologous (Rho), integrin and estrogen receptors was influenced by TRT, whereas EBRT induced RAS signaling as well as ATM, PYK and MAP kinases. Consistently with (phospho)proteomics data (Fig. 1e) WB analysis (Fig. 1f, g) confirmed elevated level of integrin receptor ligand, cysteine-rich angiogenic inducer 61 (CYR61), phosphorylation of EGFR and ERK1/2 and transcription factor c-JUN in response to TRT, whereas EBRT did not increase CYR61 protein level, ERK1/2 phosphorylation and decreased c-JUN phosphorylation. Next, to investigate whether the differences between TRT and EBRT result from different energy dose or time after irradiation, CYR61 expression was analyzed in the cells treated with 1–8 Gy and at different time points (Fig. 1h).

**Fig. 2:**
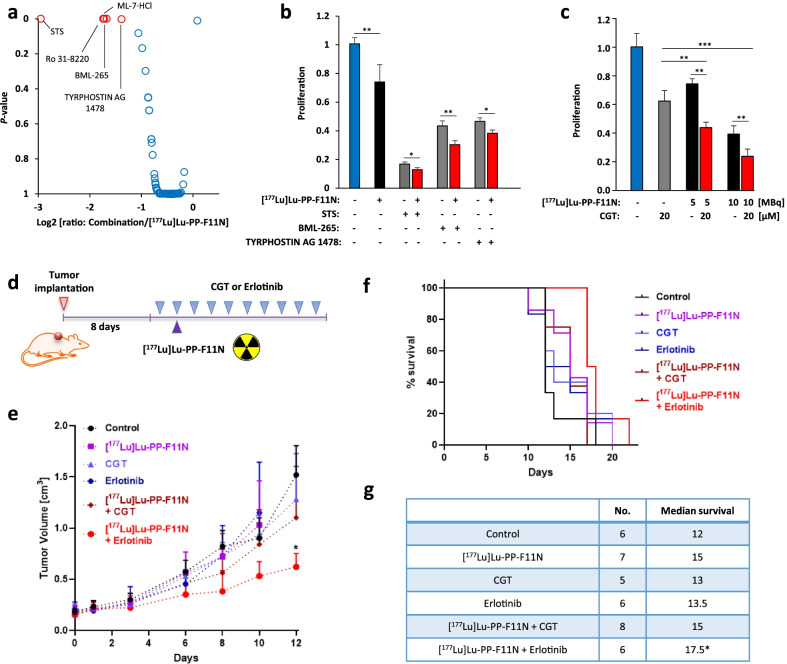
Validation of radiosensitizing targets to TRT. **a** Reduced proliferation of A431/CCKBR cells treated with kinase inhibitor library in combination with [^177^Lu]Lu-PP-F11N, shown as log2 [ratio: combinatory treatment/[^177^Lu]Lu-PP-F11N monotherapy]. Red dots represent inhibitors, which significantly increased therapeutic response to [^117^Lu]Lu-PP-F11N (*P* < 0.05). **b** Cell proliferation 48 h after treatment with 10 µM STS, BML-265 and TYRPHOSTIN AG 1478 alone or in combination with 5 MBq per ml of [^177^Lu]Lu-PP-F11N. **c** A431/CCKBR cell proliferation was analyzed 72 h after treatment with 20 µM cilengitide (CGT) and/or 5 and 10 MBq per ml of [^177^Lu]Lu-PP-F11N, as indicated. Results were assayed in triplicate and proliferation in control untreated cells was set to 1. Data represent means ± SD. **d** Experimental design of in vivo study: After implantation of A431/CCKBR cells into nude mice, 10 doses of cilengitide (CGT) or erlotinib were administrated alone or in combination with 60 kBq [^177^Lu]Lu-PP-F11N, as indicated. **e** Tumor growth curves of control and treated groups. Data represent mean ± SD. **f** Survival rates presented as Kaplan–Meier curves of control and treated mice. **g** Extended median survival in treated groups as compared to control mice. *P < 0.05, **P < 0.01, ***P < 0.001

As expected, P53 phosphorylation was increased, whereas CYR61 protein level was marginally affected by EBRT indicating that elevated CYR61 level is TRT-specific and that cancer responses differ among various types of radiations. These differences could be explained by the different ratios of various DNA strand breaks or/and types of DNA lesions or by diverse activation of DNA damage-unrelated signaling pathways. Previously, x-ray spectroscopy determined structures of DNA lesions caused by UVA light or protons and demonstrated that the cyclobutane pyrimidine dimers were almost exclusively detected in UVA-exposed samples [5]. Consequently, different types of DNA lesions can lead to the activation of different DNA repair and survival mechanisms [6] and thus, may require design of radiation-specific radiosensitizing strategies. To investigate the influence of signaling pathway activation on the survival of [^177^Lu]Lu-PP-F11N-treated cells, we employed small-molecule kinase inhibitor library. The screen identified Staurosporin (STS) as well as two EGFR inhibitors BML-265 and Tyrphostin AG 1478, which significantly enhanced therapeutic response to TRT as compared to the monotherapy and reached 12 (*P* = 0.027), 30 (*P* = 0.005) and 38% (*P* = 0.012) proliferation of control, respectively (Fig. 2a, b). STS is highly cytotoxic, whereas the combinations of [^177^Lu]Lu-PP-F11N with two other identified inhibitors Ro 31-8220 or ML-7-HCL were not superior to the monotherapy (Additional file 1: Fig. S3), and thus, their further development was not considered. Next, we analyzed the potential of cilengitide (CGT), a cyclized Arg-Gly-Glu (RGD)-containing pentapeptide, which selectively blocks activation of the αvβ3 and αvβ5 integrin receptors [7]. Combination of 5 or 10 MBq of [^177^Lu]Lu-PP-F11N with CGT significantly increased therapeutic response as compared to the monotherapy and reduced cell proliferation to 43 (*P* < 0.001) or 23% (*P* = 0.006) of control, respectively (Fig. 2c, Additional file 1: Fig. S4). In vivo, the average tumor volume, on the last day when all mice were alive, reached 1.52 cm^3^ in the control, whereas in mice treated with [^177^Lu]Lu-PP-F11N, CGT and EGFR inhibitor erlotinib alone was 1.03, 1.28 and 1.15 cm^3^, respectively (Fig. 2d, e). The tumor volume in [^177^Lu]Lu-PP-F11N-treated mice in combination with CGT was 1.15 cm^3^, whereas combination with erlotinib led to reduced tumor volume (*P* ≤ 0.002) to 0.65 cm^3^ as compared to the control. The increased survival was significant (*P* ≤ 0.026) in erlotinib and [^177^Lu]Lu-PP-F11N-treated mice (Fig. 2f, g). During therapy, there was no significant decrease in body weight (Additional file 1: Fig. S5) and evident signs of toxicity in all groups. CGT did not reveal significant therapeutic benefits when combined with TRT in our preclinical mouse model. In clinic, CGT initially showed promising activity in glioma patients in association with standard chemoradiotherapy [8], yet further study did not confirm these therapeutic benefits [9] suggesting that CGT is not optimal for clinical use. Our in vivo study validated potential of EGFR inhibition to enhance therapeutic efficacy of TRT, without severe adverse effects. Overexpression and oncogenic mutations of EGFR drive carcinogenesis, and its hyperactivation has been associated with poor prognosis and outcomes [10]. Erlotinib is approved for non-small-cell lung carcinoma (NSCLC) treatment [11] suggesting that its combination with TRT with radiolabeled minigastrin is clinically feasible. Nevertheless, radiosensitizing potential of erlotinib for other radioligands requires further investigation. In conclusion, our signaling network analysis reveals TRT-activated cellular responses, in comparison to EBRT, and identifies molecular targets for cancer radiosensitization (Additional file 3).

## Supplementary Information

**Additional file 1**. Supplementary Figure S1–S5.

**Additional file 2**. Supplementary Table S1–S7.

**Additional file 3**. Supplementary Methods.

## Data Availability

Generated phosphoproteomics and proteomics peptide and protein data analyzed during this study are included in this published article and its supplementary information files (Additional file 2: Tables S1–S7). All other data used during the current study are available from the corresponding author on reasonable request.

## Abbreviations

ATM: Ataxia–telangiectasia mutated
CGT: Cilengitide
CYR61: Cysteine-rich angiogenic inducer 61
EBRT: External beam radiation therapy
EGFR: Epidermal growth factor receptor
ERK: Extracellular signal-regulated kinase
GAPDH: Glyceraldehyde 3-phosphate dehydrogenase
HGFR: Hepatocyte growth factor receptor
LET: Linear energy transfer
MAPK: Mitogen-activated protein kinase
mTOR: Mammalian target of rapamycin
PRRT: Peptide receptor radionuclide therapy
PSMA: Prostate-specific membrane antigen
PYK: Pyruvate kinase
TGFβR: Transforming growth factor beta receptor
TRT: Targeted radionuclide therapy

## References

[1] Grzmil M, Meisel A, Behé M, Schibli R, Lewis J, Windhorst A, Zeglis B (2019). An overview of targeted radiotherapy. Radiopharmaceutical chemistry.

[2] Hennrich U, Kopka K (2019). Lutathera®: the first FDA- and EMA-approved radiopharmaceutical for peptide receptor radionuclide therapy. Pharmaceuticals (Basel).

[3] Keam SJ. Lutetium Lu 177 Vipivotide tetraxetan: first approval. Mol Diagn Ther. 2022;1–9. 10.1007/s40291-022-00594-2 PMC9099330 35553387

[4] Reubi JC, Schaer JC, Waser B (1997). Cholecystokinin(CCK)-A and CCK-B/gastrin receptors in human tumors. Cancer Res.

[5] Czapla-Masztafiak J, Szlachetko J, Milne CJ, Lipiec E, Sa J, Penfold TJ (2016). Investigating DNA radiation damage using X-ray absorption spectroscopy. Biophys J.

[6] Morgan MA, Lawrence TS (2015). Molecular pathways: overcoming radiation resistance by targeting DNA damage response pathways. Clin Cancer Res.

[7] Scaringi C, Minniti G, Caporello P, Enrici RM (2012). Integrin inhibitor cilengitide for the treatment of glioblastoma: a brief overview of current clinical results. Anticancer Res.

[8] Stupp R, Hegi ME, Neyns B, Goldbrunner R, Schlegel U, Clement PMJ (2010). Phase I/IIa study of cilengitide and temozolomide with concomitant radiotherapy followed by cilengitide and temozolomide maintenance therapy in patients with newly diagnosed glioblastoma. J Clin Oncol.

[9] Khasraw M, Lee A, McCowatt S, Kerestes Z, Buyse ME, Back M (2016). Cilengitide with metronomic temozolomide, procarbazine, and standard radiotherapy in patients with glioblastoma and unmethylated MGMT gene promoter in ExCentric, an open-label phase II trial. J Neuro-Oncol.

[10] Xu MJ, Johnson DE, Grandis JR (2017). EGFR-targeted therapies in the post-genomic era. Cancer Metast Rev.

[11] Yang ZY, Hackshaw A, Feng Q, Fu XH, Zhang YL, Mao C (2017). Comparison of gefitinib, erlotinib and afatinib in non-small cell lung cancer: a meta-analysis. Int J Cancer.

