# Supplementary material for: Comparative analysis of cancer cell responses to targeted radionuclide therapy (TRT) and external beam radiotherapy (EBRT)
Source: J Hematol Oncol. 2022 Aug 31;15:123. doi: 10.1186/s13045-022-01343-y (PMC9429584; doi:10.1186/s13045-022-01343-y)
Supplement: Supplementary file 1 — Additional file 1. Supplementary Figure S1–S5. [file 13045_2022_1343_MOESM1_ESM.pdf]

Supplementary Material

a

b

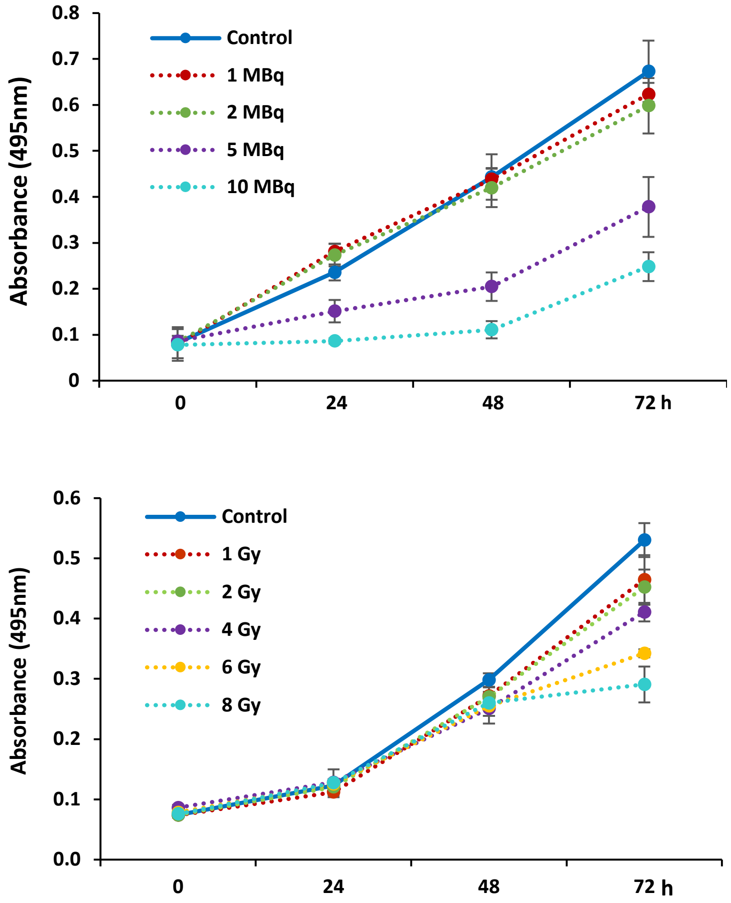

Supplementary Figure S1. Analysis of cell proliferation in response to TRT and EBRT. A431/CCKBR cell proliferation was analyzed 24, 48 and 72 h after treatment with [^{177}Lu]Lu-PP-F11N (**a**) and EBRT (**b**), as indicated. Results were assayed in triplicate and data represent means ± SD.

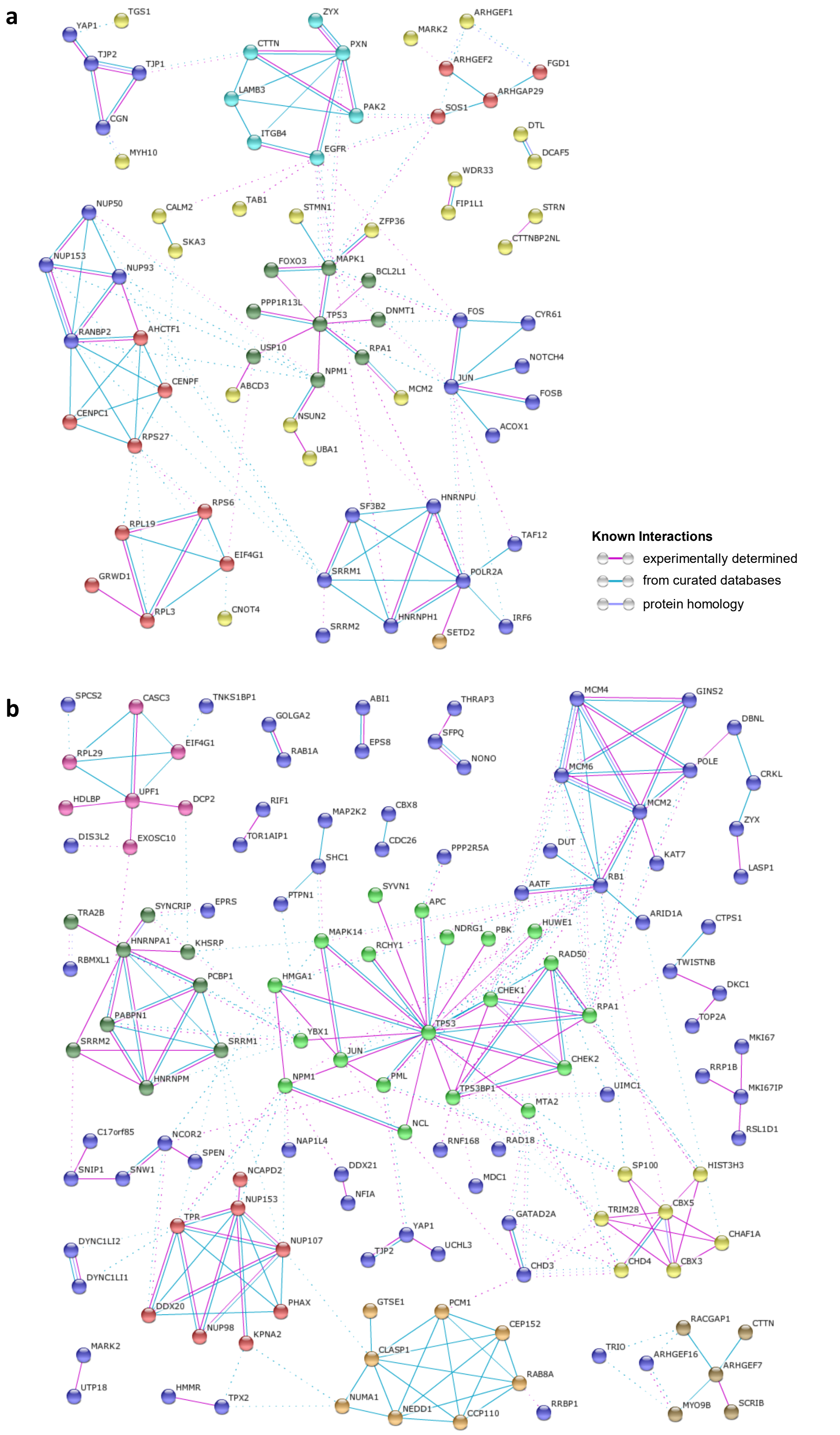

Supplementary Figure S2. Cellular responses to TRT and EBRT.

String interactom analysis of identified proteins with altered phosphorylation or protein level in response to TRT (**a**) and EBRT(**b**).

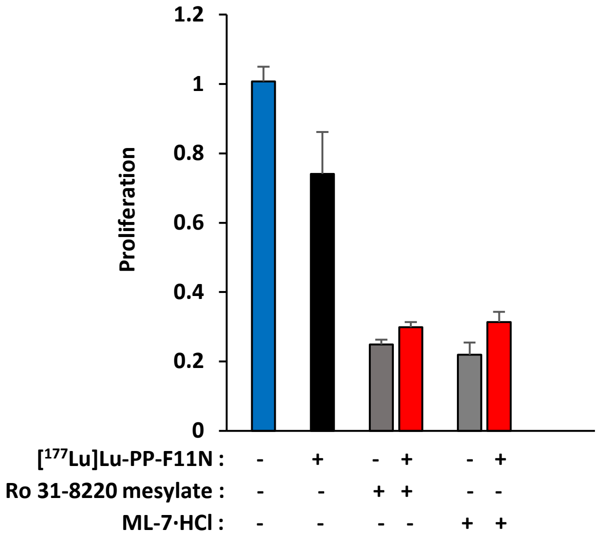

Supplementary Figure S3. **Analysis of A431/CCKBR cell proliferation in response to kinase inhibitors in combination with [^{177}Lu]Lu-PP-F11N**. Cell proliferation 48 h after treatment with 10 μM Ro 31-8220 mesylate and ML-7-HCl alone or in combination with 5 MBq per ml of [^{177}Lu]Lu-PP-F11N. Results were assayed in triplicate and data represent means ± SD.

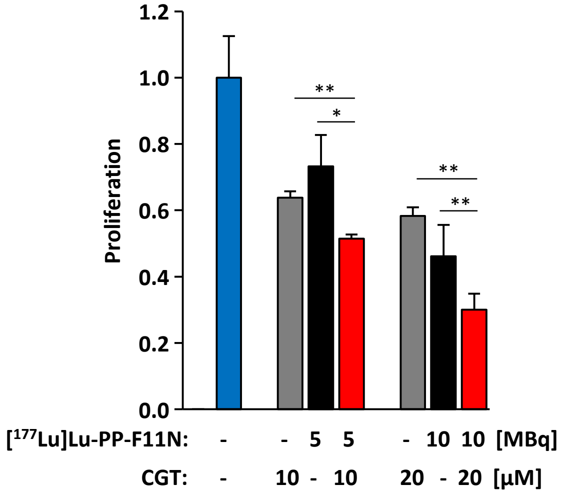

Supplementary Figure S4. **Validation of radiosensitizing targets to TRT**. A431/CCKBR cell proliferation was analyzed 48 h after treatment with 10 or 20 μM cilengitide (CGT) and/or 5 and 10 MBq per ml of [^{177}Lu]Lu-PP-F11N, as indicated. Results were assayed in triplicate and proliferation in control untreated cells was set to 1. Data represent means ± SD. *P < 0.05, **P<0.01

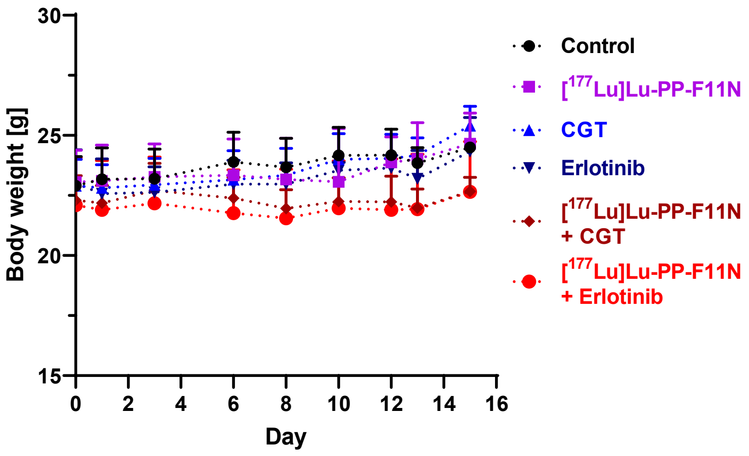

Supplementary Figure S5. **Body weight in treated mice**. Body weight of A431/CCKBR xenografted nude mice, in control and treatment groups. Values show mean ± SD.
