# Supplementary material for: Comparative analysis of cancer cell responses to targeted radionuclide therapy (TRT) and external beam radiotherapy (EBRT)
Source: J Hematol Oncol. 2022 Aug 31;15:123. doi: 10.1186/s13045-022-01343-y (PMC9429584; doi:10.1186/s13045-022-01343-y)
Supplement: Supplementary file 2 — Additional file 2. Supplementary Table S1–S7. [file 13045_2022_1343_MOESM2_ESM.pdf]

Supplementary Table S1

LOG$_2$ (ratio) ^{177}Lu-PP-F11N /CON	*P*-VALUE	SYMBOL; NAME | UniProt |	SEQUENCE WINDOW POSITION (PROBABILITY>0.5)
7.85	0.02107	HNRPU; Heterogeneous nuclear ribonucleoprotein U |Q00839|	SSS(1)PVNVK
7.17	9.71E-05	SRRM2; Serine/arginine repetitive matrix protein 2 |Q9UQ35|	RVPS(0.95)PTPAPK
6.77	3.79E-06	RSL1D1; Ribosomal L1 domain-containing protein 1 |O76021|	EKS(1)PSLGKK
6.28	0.00043	DPM1; Dolichol-phosphate mannosyltransferase subunit 1 |O60762|	ASLEVS(0.74)RSPRR
4.88	0.00062	DNTTIP2; Deoxynucleotidyltransferase terminal-interacting protein 2 |Q5QJE6|	WNNNKKS(1)PIK
4.21	0.00037	TGS1; Trimethylguanosine synthase |Q96RS0|	DRPHASGT(0.98)DGDES(0.99)EEDPPEHKPSK
4.16	0.00127	SH2D5; SH2 domain-containing protein 5 |Q6ZV89|	LGNPYCS(0.94)PTLVRK
4.11	0.00054	RPP30; Ribonuclease P protein subunit p30 |P78346|	KPRPS(1)EGDEDCLPASK
4.04	0.00174	RAB11FIP1; Rab11 family-interacting protein 1 |Q6WKZ4|	TAS(0.99)TDLK
3.83	0.00111	TGS1; Trimethylguanosine synthase |Q96RS0|	DRPHASGT(0.91)DGDES(1)EEDPPEHKPSK
3.72	0.00041	WDR33; pre-mRNA 3' end processing protein WDR33 |Q9C0J8|	DTPRPDHPPHDGHS(0.98)PASR
3.70	0.00024	EIF4ENIF1; Eukaryotic translation initiation factor 4E transporter |B1AKL4|	SS(0.82)PVESLKK
3.66	0.00411	ARHGEF1; Rho guanine nucleotide exchange factor 1 |Q92888|	QLLFPAEEDNGAGPPRDGDGVPGGGPLS(1)PAR
3.42	0.00036	DENND4C; t DENN domain-containing protein 4C |R4GN35|	RSS(0.77)LPLDHGSPAQENPESEK
3.41	0.01414	FIP1L1; Pre-mRNA 3'-end-processing factor FIP1 |Q6UN15|	ERDHS(0.99)PTPSVFNS(0.98)DEERYR
3.40	9.73E-05	TJP2; Tight junction protein ZO-2 |Q9UDY2|	SFS(1)PEER
3.40	0.00142	TRA2A; Transformer-2 protein homolog alpha |Q13595|	S(1)RS(1)HS(1)PMSNR
3.37	0.00098	TRA2A; Transformer-2 protein homolog alpha |Q13595|	S(1)RS(1)HS(0.99)PMSNR
3.21	0.00832	WARS; Tryptophan--tRNA ligase, cytoplasmic |P23381|	KLS(1)FDFQ
3.20	0.00146	TRA2A; Transformer-2 protein homolog alpha |Q13595|	S(1)RS(1)HS(0.99)PMSNR
3.12	0.00101	USP10; Ubiquitin carboxyl-terminal hydrolase 10 |Q14694|	LLS(0.99)PSNEK
3.11	5.25E-05	TP53; Cellular tumor antigen p53sp|P04637|	LMFKTEGPDS(1)D
3.10	0.00066	ITGB4; Integrin beta-4 |P16144|	DYNS(0.59)LT(0.59)RS(0.59)EHSHSTTLPR
2.98	0.00540	RPL19; Ribosomal protein L19 |J3QR09|	TLS(1)KEEETKK
2.95	0.00106	FAM83B; Protein FAM83B|Q5T0W9|	S(1)FPLFDNSK
2.91	0.00423	ANLN; Actin-binding protein anillin|Q9NQW6|	TQS(1)LPVTEK
2.68	9.87E-05	ZFP36; Tristetraprolin |P26651|	STS(0.99)LVEGR
2.37	0.01377	FOS; Proto-oncogene c-Fos|P01100|	GKVEQLS(1)PEEEEK
2.37	0.00047	PAK2; Serine/threonine-protein kinase PAK 2 |Q13177|	YLS(1)FT(1)PPEK
2.27	0.01769	ARHGEF2; Rho guanine nucleotide exchange factor |Q92974|	SES(1)LESPRGER
2.22	0.00539	EGFR; Epidermal growth factor receptor|P00533|	MHLPS(1)PTDSNFYR
2.09	0.00689	SLC20A2; Sodium-dependent phosphate transporter 2 |Q08357|	VQEAES(1)PVFK
2.09	0.00354	CCNL2; Cyclin-L2 |Q96S94|	GSKPS(0.99)PLSVK
2.06	0.00080	CCDC82; Coiled-coil domain-containing protein 82 |F5H777|	HLS(1)QEDNDLNK
2.01	0.00049	CTTNBP2NL; CTTNBP2 N-terminal-like protein |Q9P2B4|	VSSPLSPLS(1)PGIK
1.96	0.00209	SH3BP4; SH3 domain-binding protein 4 |Q9P0V3|	DNPFFRS(1)K
1.88	0.01072	ZYX; Zyxin |Q15942|	S(1)PGAPGPLTLK
1.86	0.00209	IRF6; Interferon regulatory factor 6|O14896|	LQIS(0.83)TPDIKDNIVAQLK
1.77	0.01594	EGFR; Epidermal growth factor receptor|P00533|	MHLPS(0.96)PTDS(0.99)NFYR
1.66	0.01298	AHCTF1; Protein ELYS |Q8WYP5|	STPLASPSPS(0.81)PGRS(0.91)PQR
1.66	0.00650	PFKP; ATP-dependent 6-phosphofructokinase, platelet type |Q01813|	S(1)FAGNLNTYK
1.52	0.00050	SH2D4A; SH2 domain-containing protein 4A |Q9H788|	TLS(0.99)SSAQEDIIR
1.51	0.00818	PPP1R12A; Protein phosphatase 1 regulatory subunit 12A |O14974|	TGS(0.99)YGALAEITASK
1.44	0.00740	UBAP1; Ubiquitin-associated protein 1 |Q9NZ09|	VLS(1)PPHIK
1.43	0.00091	EGFR; Epidermal growth factor receptor|P00533|	MHLPS(1)PTDSNFYR
1.43	0.00024	PPP1R12A; Protein phosphatase 1 regulatory subunit 12A |O14974|	RLAS(0.99)TSDIEEK

1.42	0.00855	NCKAP5L; Nck-associated protein 5-like |H7C1V4|	VDLEPVS(1)PR
1.36	0.00348	ABL2; Abelson tyrosine-protein kinase 2 |P42684|	VPVLIS(0.86)PTLK
1.26	7.47E-07	AKAP11; A-kinase anchor protein 11 |Q9UKA4|	S(0.99)FSEDVFQSVK
1.25	0.01012	PACSIN3; Protein kinase C and casein kinase substrate in neurons protein 3 |A0A0C4DGG1|	GGRS(0.99)PDEVTLTSIVPTR
1.20	0.00206	JUN; Transcription factor AP-1 |P05412|	LAS(1)PELER
1.11	0.00845	TNS4; Tensin-4|Q8IZW8|	ASSPHGLGSPLVAS(1)PR
1.11	0.00079	ITGB4; Integrin beta-4 |P16144|	VLS(0.99)TSSTLTR
1.09	4.57E-05	CD97; CD97 antigen|P48960|	ALRAS(0.99)ESGI
1.04	0.00424	LARP1; La-related protein 1 |Q6PKG0|	S(0.99)LPTTVPES(1)PNYR
1.04	0.00424	LARP1; La-related protein 1 |Q6PKG0|	S(0.99)LPTTVPES(1)PNYR
1.02	0.00620	BCL9L; B-cell CLL/lymphoma 9-like protein |Q86UU0|	SVS(1)VDSGEQR
1.01	0.00817	RPS27; 40S ribosomal protein S27 |P42677|	DLLHPS(1)PEEEK
0.99	0.00668	MAPK1; Mitogen-activated protein kinase 1 |P28482|	VADPDHDHTGFLT(0.98)EY(0.99)VATR
0.98	0.00579	CENPF; Centromere protein F |P49454|	SVPVNNLPERS(0.87)PTDS(0.96)PREGLR
0.97	0.00165	MARCKS; Myristoylated alanine-rich C-kinase substrate (A0A087WZH7) |P29966|	GEPAAAAAPEAGAS(1)PVEK
0.92	0.00610	FCHO2; F-BAR domain only protein 2 |Q0JRZ9|	VSIGNITLS(0.98)PAISR
0.89	0.00603	UBA1; Ubiquitin-like modifier-activating enzyme 1 |P22314|	ATLPS(0.99)PDKLPGFK
0.88	0.00540	DCAF5; DDB1- and CUL4-associated factor 5 |Q96JK2|	AEEPPS(1)PPVPK
0.87	0.00701	RBM34; RNA-binding protein 34 |A2A2V2|	S(1)VQEGENPDDGVR
0.86	0.00583	EIF4G1; Eukaryotic translation initiation factor 4 gamma 1 |Q04637|	EAALPPVS(1)PLK
0.82	0.00404	TJAP1; Tight junction-associated protein 1 |Q5JTD0|	AFVDRT(1)PPPAAVAQR
0.80	0.00079	PKP3; Plakophilin-3|Q9Y446|	ADYDTLS(0.99)LR
0.79	0.00014	NSUN2; tRNA (cytosine(34)-C(5))-methyltransferase|Q08J23|	AGEPNS(1)PDAEEANS(0.99)PDVTAGCDPAGVHPPR
0.78	0.00218	LIMA1; LIM domain and actin-binding protein 1 |Q9UHB6|	LRS(1)PPEALVQGR
0.75	0.00138	RGPD5; RANBP2-like and GRIP domain-containing protein 5/6 |Q99666|	YVASVLGLTPS(0.96)PR
0.74	0.00184	STMN1; Stathmin |P16949|	SKESVPEFPLS(1)PPK
0.64	0.00018	CCDC88A; Girdin |H0Y7K3|	FYDPS(1)PPR

Supplementary Table S1. Significantly increased phosphopeptide relative abundance in A431/CCKBR cells treated with [^{177}Lu]Lu-PP-F11N. MS2-based quantification shown as log2 ratio, *P*<0.05.

Supplementary Table S2

LOG$_2$ (ratio) ^{177}Lu-PP-F11N /CON	*P*-value	LEADING PROTEIN | ACCESSION No.|	SEQUENCE WINDOW POSITION (PROBABILITY>0.5)
-9.06	0.00323	HNRPU; Heterogeneous nuclear ribonucleoprotein U |Q00839|	SS(0.75)SPVNVK
-8.51	3.38E-05	EGFR; Epidermal growth factor receptor |P00533|	ELVEPLT(1)PS(1)GEAPNQALLR
-8.09	9.02E-05	SRRM1; Serine/arginine repetitive matrix protein 1|Q8IYB3|	T(1)PS(1)PPPR
-7.94	4.33E-05	SRRM1; Serine/arginine repetitive matrix protein 1|Q8IYB3|	T(1)PS(1)PPPR
-6.40	1.9E-05	TCOF; Treacle Ribosome Biogenesis Factor |Q13428|	SLGNILQAKPTS(0.64)SPAK
-6.11	7.02E-05	H31; Histone H3.1 |P68431|	KS(1)APATGGVK
-5.87	8.81E-05	SRRM2; Serine/arginine repetitive matrix protein 2 |Q9UQ35|	S(1)RT(1)PPSAPSQSR
-5.37	0.00043	MARK2; Serine/threonine-protein kinase MARK2 |Q7KZI7|	SVS(1)ANPK
-5.29	9.59E-06	SRC8; Src substrate cortactin |Q14247|	TQT(0.84)PPVSPAPQPTEERLPS(0.86)SPVYEDAASFK
-5.09	0.01798	SRRM2; Serine/arginine repetitive matrix protein 2 |Q9UQ35|	ELS(0.92)NSPLRENSFGS(0.99)PLEFR
-4.95	0.00011	MDC1; Mediator of DNA damage checkpoint protein 1|Q14676|	SQPPGEDS(0.99)DT(0.99)DVDDDSRPPGRPAEVHLER
-4.91	9.42E-06	TAF12; Transcription initiation factor TFIID subunit 12|Q16514|	IPGTPGAGGRLS(0.99)PENNQVLTKK
-4.58	0.00109	DKC1; H/ACA ribonucleoprotein complex subunit 4|O60832|	S(1)LPEEDVAEIQHAEEFLIKPESK
-4.55	0.00017	TR150; Thyroid hormone receptor-associated protein 3 |Q9Y2W1|	GSFS(1)DTGLGDGK
-4.47	0.00060	SRRM2; Serine/arginine repetitive matrix protein 2 |Q9UQ35|	S(1)RT(1)PPSAPSQSR
-4.45	0.00066	NIBL1; Niban-like protein 1|Q96TA1|	QVVSVVQDEEVGLPFEASPES(0.97)PPPASPDGVTEIR
-4.35	0.00076	F2Z2H1; RNA helicase Mov10l1|F2Z2H1|	MLS(1)LAAKLVAFFWR
-4.31	0.00025	PGRMC2; Membrane-associated progesterone receptor component 2|U3KQM0|	LLKPGEEPSEYT(0.99)DEEDTK
-4.28	0.00489	EGFR; Epidermal growth factor receptor |P00533|	YSSDPTGALTEDSIDDTFLPVPEY(0.97)INQSVPK
-3.99	0.00248	A0A087WZ13; Ribonucleoprotein PTB-binding 1 |A0A087WZ13|	AADVSVTHRPPLS(1)PK
-3.91	0.00024	NELFE; Negative elongation factor E |P18615|	SGAHSS(0.67)ASPPR
-3.81	0.00028	FOXO3; Forkhead box protein O3 |O43524|	AEAPAS(0.99)PAPLSPLEVELDPEFEPQSRPR
-3.73	0.00227	SOS1; Son of sevenless homolog 1 G5E9C8; |G5E9C8|	RRPESAPAESS(0.81)PSK
-3.70	0.00322	POLR2A; DNA-directed RNA polymerase subunit |A0A0C4DGZ0|	YSPTS(0.99)PTYSPTSPK
-3.64	0.00226	SART3; Squamous cell carcinoma antigen recognized by T-cells 3 |Q15020|	ATAAETS(0.99)ASEPEAESK
-3.62	4.07E-05	SF3B2; Splicing factor 3B subunit 2|Q13435|	SSLGQS(1)AS(1)ETEEDTVSVSKK
-3.61	0.00041	NOTCH4; Neurogenic locus notch homolog protein 4 |Q99466|	LEDPCPPS(1)FCS(1)K
-3.53	0.00011	NOTCH4; Neurogenic locus notch homolog protein 4 |Q99466|	LEDPCPPS(1)FCS(1)K
-3.50	0.00038	ZN800; Zinc finger protein 800|Q2TB10|	VKQDSESPKSTS(0.71)PSAAGGQQK
-3.49	0.00188	RPA1; Replication protein A 70 kDa DNA-binding subunit|P27694|	GARVS(1)DFGGR
-3.46	0.00357	CHAP1; Chromosome alignment-maintaining phosphoprotein 1 |Q96JM3|	KPGPPLS(1)PEIRS(1)PAGS(1)PELR
-3.43	0.00308	CHAP1; Chromosome alignment-maintaining phosphoprotein 1 |Q96JM3|	KPGPPLS(1)PEIRS(1)PAGS(1)PELR
-3.38	8.86E-05	CCDC6; Coiled-coil domain-containing protein 6 |Q16204|	SGGIVIS(0.99)PFRLEELTNR
-3.33	0.00137	RAVER1; Ribonucleoprotein PTB-binding 1|A0A087WZ13|	AADVSVTHRPPLS(0.99)PKS(1)GAEVEAGDAAER
-3.31	0.00087	LAD1; Ladinin-1|O00515|	RATASEQPLAQEPPASGGS(0.74)PATTK
-3.30	0.00036	YAP1; Transcriptional coactivator YAP1 |P46937|	NINPSTANS(0.99)PK
-3.22	0.02100	RRP1B; Ribosomal RNA processing protein 1 homolog B |Q14684|	VAFDPEQKPLHGVLKT(0.99)PTSSPASSPLVAK
-3.19	0.00138	HDAC7; Histone deacetylase 7|Q8WUI4|	TVS(1)EPNLK
-3.16	0.00042	MECP2; Methyl-CpG-binding protein 2|P51608|	AETSEGSGSAPAVPEASAS(0.92)PK
-3.12	0.00317	SLK; STE20-like serine/threonine-protein kinase|Q9H2G2|	SNS(0.99)EDKLNSK
-3.11	0.00408	CHAP1; Chromosome alignment-maintaining phosphoprotein 1 |Q96JM3|	KPGPPLS(1)PEIRS(1)PAGS(1)PELR
-3.03	0.00148	ARHG5; Rho guanine nucleotide exchange factor 5|Q12774|	TTPQQGASGPGRS(0.94)PVGQAR
-3.02	0.00075	RAD18; E3 ubiquitin-protein ligase RAD18|Q9NS91|	NHLLQFALES(1)PAKS(0.98)PASSSSK
-3.00	0.00021	RPA34; DNA-directed RNA polymerase I subunit RPA34 |O15446|	ILEGPQQSLSGS(0.78)PLQPIPAS(1)PPPQIPPGLRPR
-2.96	0.00131	NUCKS; Nuclear ubiquitous casein and cyclin-dependent kinase substrate 1 |Q9H1E3|	S(0.99)GKNSQEDSEDSEDKDVK
-2.95	0.00126	CCKBR; Gastrin/cholecystokinin type B receptor |E9PIC8|	CRPETGAVGEDSDGCY(1)VQLPR

Supplementary Table S2

-2.91	0.00112	ZMYND8; Protein kinase C-binding protein 1 |H7C4X9|	TPPSTTVGSHS(0.92)PPETPVLTR
-2.90	0.00023	DCP1A; mRNA-decapping enzyme 1A |Q9NPI6|	HAPTYTIPLS(0.84)PVLS(0.94)PTLPAEAPTAQVPPSLPR
-2.85	0.00733	CENPC; Centromere protein C |Q03188|	ILATDVSSKNT(0.96)PDSK
-2.85	0.00059	KMT2A; Histone-lysine N-methyltransferase 2A |Q03164|	ALSSAVQASPTS(0.81)PGGSPSS(0.55)PSSGQR
-2.85	0.00429	RPA34; DNA-directed RNA polymerase I subunit RPA34 |O15446|	ILEGPQQSLSGS(0.96)PLQPIPASPPPQIPPGLRPR
-2.83	8.03E-06	CGN; Cingulin |A6PVU7|	SHS(0.98)QASLAGPGPVDPSNR
-2.79	0.00578	NUP50; Nuclear pore complex protein Nup50 |Q9UKX7|	KVDSSVLGSLSSVPLTGFSFS(0.98)PGNSSLFGK
-2.79	0.00074	NUP153; Nuclear pore complex protein Nup153|P49790|	CIACQAAKLS(1)PR
-2.76	0.00543	PAXB1; PAX3- and PAX7-binding protein 1 |Q9Y5B6|	LVREDENDAS(1)DDEDDDEK
-2.72	0.00290	RAD18; E3 ubiquitin-protein ligase RAD18|Q9NS91|	NHLLQFALES(1)PAK
-2.72	0.00130	RFC1; Replication factor C subunit 1 |P35251|	NKPLS(1)PIKLT(0.99)PTSVLDYFGTGSVQR
-2.72	0.00243	ABCD3; ATP-binding cassette sub-family D member 3 |P28288|	S(1)GKPPLQNNEK
-2.65	0.00052	CNTROB; Centrobin |I3L465|	AQLYLPSTS(0.63)PPHEGLDGFAQELSR
-2.61	0.00012	SSB; Lupus La protein |P05455|	S(0.92)PSKPLPEVTDEYKNDVK
-2.58	0.00199	PPP1R13L; RelA-associated inhibitor|Q8WUF5|	HGGPGPGGPEPELS(0.99)PITEGSEAR
-2.57	0.00038	RFC1; Replication factor C subunit 1 |P35251|	SSPAKKESVS(0.97)PEDSEK
-2.57	0.00658	DCP1A; mRNA-decapping enzyme 1A |Q9NPI6|	HAPTYTIPLS(0.88)PVLS(0.87)PTLPAEAPTAQVPPSLPR
-2.56	0.00054	DNMT1; DNA (cytosine-5)-methyltransferase 1 |P26358|	SDGEAKPEPS(1)PS(1)PR
-2.56	0.00053	RFC1; Replication factor C subunit 1 |P35251|	NKPLS(1)PIK
-2.52	0.00992	MINK1; Misshapen-like kinase 1 |Q8N4C8|	NRVGVSSKPDSS(0.95)PVLSPGNK
-2.51	0.00185	BCLF1; Bcl-2-associated transcription factor 1 |Q9NYF8|	SGSS(0.86)PKWTHDK
-2.45	0.01394	CNOT4; CCR4-NOT transcription complex subunit 4 |O95628|	ELSVQDQPSLS(0.92)PTSLQNSSSHTTTAK
-2.45	0.00210	KMT2A; Histone-lysine N-methyltransferase 2A|Q03164|	ALSSAVQASPTS(0.63)PGGSPSS(0.65)PSSGQR
-2.44	0.00233	RHAGAP29; Rho GTPase-activating protein 29 |Q52LW3|	SLDSESIS(0.99)PGDFHR
-2.44	0.00620	RS6; 40S ribosomal protein S6 |P62753|	RLS(1)S(1)LRAS(0.96)TSK
-2.43	0.00442	PKP3; Plakophilin-3 |Q9Y446|	LSS(0.99)GFDDIDLPSAVK
-2.41	0.00600	CIC; Protein capicua homolog |I3L2J0|	ACTGLSGPGSGSKS(0.64)PPATR
-2.34	0.00334	DYNC1LI2; Cytoplasmic dynein 1 light intermediate chain 2 |O43237|	KTGS(0.94)PGS(0.99)PGAGGVQSTAKK
-2.29	0.00174	KIF1B; Kinesin-like protein KIF1B |A0A087WWA3|	AS(0.5)S(0.5)PCPEFEQFQIVPAVETPYLAR
-2.27	0.00068	CCDC9; Coiled-coil domain-containing protein 9 |Q9Y3X0|	EGAASPAPETPQPTS(0.69)PETSPK
-2.25	0.00275	DTL; Denticleless protein |F5GZ90|	TPSSS(0.80)PPIT(0.99)PPASETK
-2.25	0.01660	RL1D1; Ribosomal L1 domain-containing protein 1 |O76021|	KS(1)PAKS(0.99)PNPSTPR
-2.22	0.00771	KIF1B; Kinesin-like protein KIF1B |A0A087WWA3|	AS(0.5)S(0.5)PCPEFEQFQIVPAVETPYLAR
-2.19	0.00429	CDR2; Cerebellar degeneration-related protein 2|Q01850|	SSS(0.85)ETILSSLAGSDIVK
-2.17	0.00033	RHPN2; Rhophilin-2 |Q8IUC4|	SAS(0.95)TLCLPSVGAARPQVK
-2.07	0.01372	RBM34; RNA-binding protein |A2A2V2|	SVQEGENPDDGVRGS(0.99)PPEDYR
-2.05	0.00085	MAGI3; Membrane-associated guanylate kinase |Q5TCQ9|	IFYGEKQPEDDSSQAFISTQNGS(0.99)PR
-2.043	9.9E-05	SRCAP; Helicase SRCAP |A0A0A0MS59|	GAASTLVPGVSETSAS(0.59)PGSPSVR
-1.93	0.01488	CLDN4; Claudin-4 |O14493|	SAAASNY(1)V
-1.91	0.00112	ATP13A1; Manganese-transporting ATPase 13A1 |Q9HD20|	DS(0.98)PTLSNSGIR
-1.87	0.01166	ZC3HC1; Nuclear-interacting partner of ALK |C9J0I9|	SWDSSS(0.75)PVDRPEPEAAS(0.71)PTTR
-1.86	0.0146	PDLIM2; PDZ and LIM domain protein 2 |Q96JY6|	AGS(1)PFS(0.99)PPPSSSSLTGEAAISR
-1.84	0.00059	SETD2; Histone-lysine N-methyltransferase SETD2 |H7BZ93|	LNDS(0.99)PTLK
-1.82	0.01259	ZN318; Zinc finger protein 318 |Q5VUA4|	RSS(0.8)PPPPPSGSSSRTPAR
-1.79	0.01615	TJP1; Tight junction protein ZO-1 |Q07157|	LEEPTPAPSTSYS(0.98)PQADSLR
-1.73	0.00989	DNMT1; DNA (cytosine-5)-methyltransferase 1|P26358|	SDGEAKPEPS(1)PS(1)PR
-1.72	0.00104	RPS6; 40S ribosomal protein S6|P62753|	RLS(1)S(1)LRASTSK

Supplementary Table S2

-1.72	0.01616	MCM2; DNA replication licensing factor MCM2 |P49736|	AIPELDAYEAEGLALDDEDVEELT(0.55)ASQREAAER
-1.69	0.01214	RPS6; 40S ribosomal protein S6|P62753|	LSSLRASTSKS(0.96)ESS(0.69)QK
-1.67	0.01230	LRWD1; Leucine-rich repeat and WD repeat-containing protein 1 |Q9UFC0|	ACAS(0.9)PSAQVEGS(1)PVAGSDGSQPAVK
-1.65	0.01201	RPL3; 60S ribosomal protein L3 |P39023|	S(1)LLVQTK
-1.61	0.00736	RSL1D1; Ribosomal L1 domain-containing protein 1 |O76021|	KS(1)PAKS(0.99)PNPSTPR
-1.59	0.00030	UBE2O; (E3-independent) E2 ubiquitin-conjugating enzyme |Q9C0C9|	IMSCS(0.97)PDTQCSR
-1.56	0.01326	AFF4; AF4/FMR2 family member 4 |Q9UHB7|	ELLSPLS(0.99)EPDDRYPLIVK
-1.51	0.00699	PAX; Paxillin |P49023|	DGGRS(0.79)SPGGQDEGGFMAQGK
-1.47	0.01439	BAG3; BAG family molecular chaperone regulator 3 |O95817|	SST(0.94)PLHS(PS(0.93)PIR
-1.42	0.0092	NPM1; Nucleophosmin |P06748|	S(1)APGGGSKVPQK
-1.33	0.01066	HNRNPH1; Heterogeneous nuclear ribonucleoprotein H |P31943|	EGRPSGEAFVELES(1)EDEVK
-1.30	0.00048	DDX39A; ATP-dependent RNA helicase DDX39A |O00148|	NRS(0.99)FSLK
-1.14	0.00396	H14; Histone H1.4 |P10412|, H15; Histone H1.5 |P16401|, H13; Histone H1.3|P16402|, H12; Histone H1.2 |P16403|, H11; Histone H1.1|Q02539|	GTGAS(0.8)GSFK
-1.11	0.00914	FANCI; Fanconi anemia group I protein|Q9NVI1|	TIETS(0.95)PSLSR
-1.11	0.00589	CALM1; Calmodulin|P62158|	VFDKDGNGY(1)ISAAELR
-1.10	0.00953	FGD1; FYVE, RhoGEF and PH domain-containing protein 1 |P98174|	RGS(0.7)GSALGGPLDPQFVGPSDTSLGAAPGHR
-1.06	0.00434	H15; Histone H1.5 |P16401|	SETAPAETAT(1)PAPVEKS(1)PAKK
-1.06	0.00434	H15; Histone H1.5 |P16401|	SETAPAETAT(1)PAPVEKS(1)PAKK
-0.97	0.00071	MYBBP1A; Myb-binding protein 1A |Q9BQG0|	SPS(1)LLQSGAK
-0.89	0.00044	SKA3; Spindle and kinetochore-associated protein 3|Q8IX90|	NSVHEQEAINS(1)DPELSNCENFQK
-0.76	0.00137	NUP93; Nuclear pore complex protein Nup93 |Q8N1F7|	GTS(0.98)PSSSSRPQR
-0.74	0.002855	ACOX1; Peroxisomal acyl-coenzyme A oxidase 1 |Q15067|	DSASFNPELLTHILDGS(1)PEK
-0.64	0.002831	RANBP2; E3 SUMO-protein ligase RanBP2 |P49792|	NADSEIKHST(0.97)PS(0.94)PTR

Supplementary Table S2. Significantly decreased phosphopeptide relative abundance in A431/CCKBR cells treated with [^{177}Lu]Lu-PP-F11N. MS2-based quantification shown as log2 ratio, *P*<0.05.

Supplementary Table S3

LOG_2 (ratio) EBRT / CONTROL	*P*-VALUE	SYMBOL; NAME | UniProt |	SEQUENCE WINDOW POSITION (PROBABILITY>0.5)
8.69	0.00010	NUCKS1; Nuclear ubiquitous casein and cyclin-dependent kinase substrate 1 |Q9H1E3|	IRS(0.5)S(0.5)PREAK
6.74	0.00033	EIF4G1; Eukaryotic translation initiation factor 4 gamma 1 |E7EX73|	SSLS(0.99)RERGEK
6.19	0.00053	EIF4G1; Eukaryotic translation initiation factor 4 gamma 1 |E7EX73|	RVVQRS(0.69)SLSR
6.15	0.00013	RIF1; Telomere-associated protein RIF1 |Q5UIP0|	RSEVVESTTES(1)QDK
6.07	0.00043	SRRM2; Serine/arginine repetitive matrix protein 2 |Q9UQ35|	S(1)KT(0.95)PSRQSHSS(0.95)S(0.64)SP
5.88	7.13E-05	SRRM2; Serine/arginine repetitive matrix protein 2 |Q9UQ35|	S(0.99)KT(0.99)PSRQSHSSSSPHPK
5.77	4.83E-05	ZNF638; Zinc finger protein 638 |Q14966|	AVIVS(0.81)SPK
5.74	0.00073	RPL29; 60S ribosomal protein L29 |P47914|	RTQAPTKAS(1)E
5.68	0.00039	SRRM2; Serine/arginine repetitive matrix protein 2 |Q9UQ35|	PES(1)PKPAPAPGSHREIS(0.98)S(0.68)S
5.68	1.51E-05	ZNF318; Zinc finger protein 318 |Q5VUA4|	RAS(1)PS(1)PPRGR
5.61	0.00021	IWS1; Protein IWS1 homolog |Q96ST2|	KFHS(1)S(1)DS(1)EEEEHKK
5.60	0.00046	ZNF318; Zinc finger protein 318 |Q5VUA4|	RAS(1)PS(1)PPRGR
5.60	0.00051	CRKL; Crk-like protein |P46109|	LVRSS(0.73)PHGK
5.41	0.00058	SNIP1; Smad nuclear-interacting protein 1 |Q8TAD8|	RTS(0.96)NERPGSGQGQGR
5.40	0.00054	CRKL; Crk-like protein |P46109|	LVRS(0.5)S(0.5)PHGK
4.65	0.01448	MDC1; Mediator of DNA damage checkpoint protein 1 |Q14676|	DTQRGEPEGGS(1)QDQK
4.64	0.00868	DUT; Deoxyuridine 5'-triphosphate nucleotidohydrolase, mitochondrial |H0YNJ9|	PCS(1)EETPAIS(1)PSK
4.55	0.00190	MAP4; Microtubule-associated protein |E7EVA0|	RAS(0.99)PSKPASAPASR
4.51	0.00010	RIF1; Telomere-associated protein RIF1 |Q5UIP0|	NVS(1)QESLETK
4.51	0.00023	POLE; DNA polymerase epsilon catalytic subunit A |F5H1D6|	IHCGLQDS(1)QK
4.43	0.00235	ZZZ3; ZZ-type zinc finger-containing protein 3 |Q8IYH5|	RAAPTRGS(0.99)PTK
4.35	7.60E-06	ADAR; Double-stranded RNA-specific adenosine deaminase |P55265|	TMLLLS(0.87)RSPEAQPK
4.34	0.00026	OXSR1; Serine/threonine-protein kinase OSR1 |O95747|	TAQALSSGSGS(0.88)QETK
4.28	0.00601	MCM4; DNA replication licensing factor MCM4 |P33991|	S(0.5)S(0.5)PAST(0.73)PSRRGSR
4.28	0.00270	PRPF38B; Pre-mRNA-splicing factor 38B |Q5VTL8|	RRS(1)QS(1)IEQES(1)QEK
4.13	0.00139	SP100; Nuclear autoantigen Sp-100 |P23497|	DTTSDKDDSLGS(1)QQTNEQCAQK
4.13	0.00013	RB1; Retinoblastoma-associated protein |P06400|	LQNNHTAADMY(0.72)LSPVRS(0.96)PK
4.06	0.00192	SSRM1; Serine/arginine repetitive matrix protein 1 |A9Z1X7|	QRSS(0.7)PVTK
4.04	0.00032	DSG2; Desmoglein-2|Q14126|	AAS(1)YTEEDENHTAK
3.89	0.00023	POLR2A; DNA-directed RNA polymerase II subunit RPB1 |A0A0C4DGZ0| |P24928|	LLVDS(1)NNPKIK
3.85	0.01577	SRRM2; Serine/arginine repetitive matrix protein 2 |Q9UQ35|	YSHSGSS(0.88)SPDTK
3.83	0.00087	CHEK1; Non-specific serine/threonine protein kinase |J3KN87|	VTS(1)GGVSESPSGFSK
3.82	0.00011	ENSA; Alpha-endosulfine |O43768|	S(1)QKQEEENPAEETGEEK
3.79	0.00487	RIF1; Telomere-associated protein RIF1 |Q5UIP0|	NVS(0.99)QES(0.93)LETKEEKPEETPK
3.72	0.00384	NFIC; Nuclear factor 1 C-type|P08651|	KTEMDKS(1)PFNSPSPQDSPR
3.64	0.00235	RNF181; E3 ubiquitin-protein ligase RNF181 |C9J1C6|	TVIRGS(0.99)QAELK
3.59	0.00075	USP1; Ubiquitin carboxyl-terminal hydrolase 1 |O94782|	RALDFTDS(1)QENEEK
3.54	0.00098	TP53; Cellular tumor antigen p53 |P04637|	MEEPQSDPSVEPPLS(0.98)QETFSDLWK
3.53	0.00055	RNF168; E3 ubiquitin-protein ligase RNF168 |Q8IYW5|	VAAERRAS(1)EEEENK
3.52	0.00228	CHAF1A; Chromatin assembly factor 1 subunit A |Q13111|	GLLSNHTGS(0.98)PR
3.49	0.00671	PATJ; InaD-like protein |Q8NI35|	VPDS(1)PENELK
3.45	0.00288	DYNC1LI2; Cytoplasmic dynein 1 light intermediate chain 2 |O43237|	ASESPARGPS(0.6)GSPR
3.40	0.00082	UTP14A; U3 small nucleolar RNA-associated protein 14 homolog A |Q9BVJ6|	DSGS(1)QEVLSELR
3.39	0.00285	CEP152; Centrosomal protein of 152 kDa |O94986|	NVS(1)PEFVPCEGEGGFGLHK
3.35	0.01188	RAD50; DNA repair protein RAD50 |Q92878|	LFDVCGS(1)QDFESDLDRLKEEIEK
3.31	0.00058	DDX47; Probable ATP-dependent RNA helicase DDX47 |Q9H0S4|	AAPEEHDSPTEAS(1)QPIVEEEETK
3.23	0.00036	SLC4A1AP; Kanadaptin |A0A087X0M4|	ETQTHENMS(1)QLS(1)EEEQNK
3.21	0.00076	NTHL1; Endonuclease III-like protein 1 |P78549|	SRS(0.99)LGPGAGPR
3.20	0.00101	KHSRP; Far upstream element-binding protein 2 |Q92945|	VQIS(1)PDSGGLPER
3.17	0.00101	TWISTNB; DNA-directed RNA polymerase I subunit RPA43 |Q3B726|	KKDPETYEVDS(0.83)GTTK
3.08	0.00075	UIMC1; BRCA1-A complex subunit RAP80 |Q96RL1|	MSEQEAREVNS(1)QEEEEEELLRK
3.03	0.00187	SIPA1L1; Signal-induced proliferation-associated 1-like protein |O43166|	HSAS(1)PVVFTSAR
3.03	0.00623	PHLDB1; Pleckstrin homology-like domain family B member 1 |Q86UU1|	SES(0.99)PRLSRK
3.01	0.00442	NCOR2; Nuclear receptor corepressor 2 |C9J7T7|	SRS(0.99)PAPPADK
2.93	0.00016	HMGA1; High mobility group protein HMG-I/HMG-Y |P17096|	KLEKEEEEGIS(1)QES(1)S(1)EEEQ

Supplementary Table S3

2.92	0.00019	THRAP3; Thyroid hormone receptor-associated protein 3 |Q9Y2W1|	SSS(0.98)KDS(0.76)RPSQAAGDNQGDE
2.89	0.00044	GINS2; DNA replication complex GINS protein PSF2 |Q9Y248|	LRTNLQPLESTQS(1)QDF
2.85	0.01394	THRAP3; Thyroid hormone receptor-associated protein 3 |Q9Y2W1|	SSS(0.98)KDS(0.76)RPSQAAGDNQGDE
2.83	9.40E-05	MAP2K2; Dual specificity mitogen-activated protein kinase kinase 2 |P36507|	ELEAIFGRPVVDGEEGEPHSIS(0.8)PR
2.76	0.00082	ARID1A; AT-rich interactive domain-containing protein 1A |O14497|	SNS(1)VGIQDAFNDGSDSTFQK
2.71	0.00647	ZSWIM8; Zinc finger SWIM domain-containing protein 8 |H0YCW2|	GWGS(1)PGRPK
2.67	0.00671	HNRNPM; Heterogeneous nuclear ribonucleoprotein M |P52272|	MGS(1)GIER
2.63	0.00074	RCHY1; RING finger and CHY zinc finger domain-containing protein 1 |Q96PM5|	RIS(1)LDQQ
2.61	0.00658	RAD51AP1; RAD51-associated protein 1 |F5H1Y0|	ELPTVTTNVQNS(1)QDK
2.57	0.00702	TRIM28: Transcription intermediary factor 1-beta |Q13263|	S(1)GEGEVSGLMR
2.57	0.00963	FAM83G; Protein FAM83G |A6ND36|	LLPDPGS(1)PR
2.55	0.00124	TCOF1; Treacle protein |Q13428|	GSLGS(1)QGAKDEPEEELQK
2.54	0.01405	MCM6; DNA replication licensing factor MCM6 |Q14566|	EIESEIDS(1)EEELINKK
2.53	0.01208	PPP2R5A; Serine/threonine-protein phosphatase 2A 56 kDa regulatory subunit alpha isoform |Q15172|	RSQGS(0.91)S(0.92)QFRSQGSQAELHPL
2.50	0.00047	C2CD5; C2 domain-containing protein 5 |Q86YS7|	SLQRAST(0.54)DNEELLQFPLELCSDSLPS
2.48	0.01367	CAST; Calpastatin |A0A0C4DGB5|	SESELIDELS(0.99)EDFDRSECK
2.47	0.00940	PHAX; Phosphorylated adapter RNA export protein |Q9H814|	ETFASDTNEALASLDES(0.99)QEGHAEAK
2.47	0.00555	RREB1; Ras-responsive element-binding protein 1 |Q92766|	VTENGGSPQGIKS(0.77)PSKPPGPNR
2.42	0.00076	YBX1; Nuclease-sensitive element-binding protein 1 |P67809|	NYQQNYQNSES(0.97)GEK
2.41	0.00179	SNW1; SNW domain-containing protein 1 |Q13573|	ALTSFLPAPTQLS(1)QDQLEAEEK
2.38	0.00867	CCP110; Centriolar coiled-coil protein of 110 kDa |O43303|	LPSPEPSMS(0.96)PK
2.35	0.01272	NDRG1; Protein NDRG1|Q92597|	SRS(0.59)HT(0.54)SEGAHLDITPNSGAA
2.34	0.00305	YAP1: Transcriptional coactivator YAP1 |P46937|	AHS(0.91)SPASLQLGAVSPGTLTPTGVVS
2.33	0.00089	RALGPS2; Ras-specific guanine nucleotide-releasing factor RalGPS2 |Q86X27|	SVAAEGALLPQT(0.91)PPSPR
2.32	0.00432	CHEK2; Serine/threonine-protein kinase Chk2 |O96017|	FAIGS(1)AREADPALNVETEIEILK
2.32	0.00082	CHD4; Chromodomain-helicase-DNA-binding protein 4 |F5GWX5|	IEENS(1)LKEEESIEGEK
2.25	0.00847	TP53BP1; Tumor suppressor p53-binding protein 1 |Q12888|	LVSPETEAS(1)EESLQFNLEK
2.23	0.00668	MAPK14; Mitogen-activated protein kinase 14 |Q16539|	S(1)QERPTFYR
2.17	0.00801	SYNCRIP; Heterogeneous nuclear ribonucleoprotein Q |O60506|	YGGPPPDSVYSGQQPS(0.93)VGTEIFVG
1.98	0.01174	CDC26; Anaphase-promoting complex subunit CDC26 |Q8NHZ8|	QKEDVEVVGGS(1)DGEGAIGLSSDPK
1.94	0.00812	MYPN; Myopalladin |A0A087WX60|	TPVDES(1)DDEIQHDEIPTGK
1.83	0.00705	PABPN1; Polyadenylate-binding protein 2 |Q86U42|	APGPGPGSGAPGS(1)QEEEEEPGLVEGD
1.82	0.00629	CBX3; Chromobox protein homolog 3 |Q13185|	LTWHS(0.99)CPEDEAQ
1.76	0.01134	HMGA1; High mobility group protein HMG-I/HMG-Y |P17096|	KQPPVSPGTALVGS(1)QK
1.74	0.01094	TRIO; Triple functional domain protein |E7EPJ7|	GGSFWSSIPAS(0.90)PASRPGSFTFPGDS
1.68	0.00295	UTP14A; U3 small nucleolar RNA-associated protein 14 homolog A |Q9BVJ6|	RSELSQDAEPAGS(1)QETK
1.53	0.00778	LRBA; Lipopolysaccharide-responsive and beige-like anchor protein |E9PEM5|	ANLPTELQDNSLS(0.93)PAASEAGEK
1.28	0.00377	MCM2; DNA replication licensing factor MCM2|P49736|	EGLALDDEDVEELTAS(0.99)QREAAER
0.97	0.00416	BCLAF1; Bcl-2-associated transcription factor 1 |Q9NYF8|	S(1)QEEPKDTFEHDPSESIDEFNK
0.71	0.00061	UPF1; Regulator of nonsense transcripts 1 |Q92900|	SQIDVALS(0.99)QDSTYQGER

Supplementary Table S3. Significantly increased phosphopeptide relative abundance in A431/CCKBR cells after EBRT. MS2-based quantification shown as log2 ratio, $P<0.05$.

Supplementary Table S4

LOG$_2$ (ratio) EBRT / CONTROL	*P*-VALUE	SYMBOL; NAME |UniProt|	SEQUENCE WINDOW POSITION (PROBABILITY>0.5)
-7.62	1.43E-05	ARHGEF16; Rho guanine nucleotide exchange factor 16|Q5VV41|	AQRHSDS(0.96)SLEEK
-7.60	1.8E-06	HIST3H3; Histone H3.1t |Q16695|	VARKS(1)APATGGVK
-6.77	0.00037	NOLC1; Nucleolar and coiled-body phosphoprotein 1|Q14978|	VAGGAAPSKPAS(1)AKK
-6.53	0.01849	NPM1; Nucleophosmin|P06748|	T(1)PKGPSSVEDIK
-6.14	4.61E-05	SRRM2; Serine/arginine repetitive matrix protein 2|Q9UQ35|	SST(0.98)PPGESYFGVSSLQLK
-6.01	1.46E-05	NPM1; Nucleophosmin|P06748|	T(1)PKT(1)PKGPSSVEDIK
-5.98	4.31E-05	EPRS; Bifunctional glutamate/proline--tRNA ligase|P07814|	EYIPGQPPLSQSSDS(0.84)SPTRNSEPAG
-5.73	0.00030	UBAP2L; Ubiquitin-associated protein 2-like|Q14157|	NPSDSAVHS(0.98)PFTK
-5.41	0.00027	RSL1D1; Ribosomal L1 domain-containing protein 1|O76021|	AAESET(1)PGKS(1)PEK
-5.32	0.01799	NCL; Nucleolin|P19338|	ALVAT(1)PGKK
-5.30	0.00032	JUN; Transcription factor AP-1|P05412|	LAS(1)PELER
-5.28	0.00050	HIST1H1B; Histone H1.5|P16401|	SETAPAETAT(1)PAPVEKS(1)PAK
-5.19	6.3E-05	RPRD2; Regulation of nuclear pre-mRNA domain-containing protein 2|Q5VT52|	NTGVSPASRPSPGTPTS(0.81)PSNLTSGL
-5.14	1.03E-05	MKI67; Proliferation marker protein Ki-67|P46013|	RES(1)VNLGK
-5.06	0.01149	RSRC2; Arginine/serine-rich coiled-coil protein 2|Q7L4I2|	AAS(0.99)DTERDGLAPEK
-5.05	0.00012	DIS3L2; DIS3-like exonuclease 2|Q8IYB7|	GVSAVAGPHDIGAS(1)PGDKK
-5.00	1.49E-05	MKI67; Proliferation marker protein Ki-67|P46013|	GRDVESVQT(0.99)PSK
-4.96	0.00020	PRCC; Proline-rich protein PRCC|Q92733|	KPSDGS(0.99)PDTKPSR
-4.82	0.00404	MTA2; Metastasis-associated protein MTA2|O94776|	GHLSRPEAQSLS(0.99)PYTTSANR
-4.74	0.00054	ESF1; ESF1 homolog|Q9H501|	ALAEEAS(1)EEELPS(1)DVDLNDPYFAEEV
-4.72	2.4E-05	CTPS1; CTP synthase 1 |P17812|	DTYSDRSGS(0.88)SS(0.80)PDSEITELK
-4.70	0.00012	DTNB; Dystrobrevin|E7EVB6|	LRLEHEQASQPT(1)PEK
-4.57	0.00079	LASP1; LIM and SH3 domain protein 1 |Q14847|	GFSVVADT(1)PELQR
-4.53	0.00048	SPAG1; Sperm-associated antigen 1 |Q07617|	GQTPEAGADKRS(1)PR
-4.53	0.00032	A0A087WUU9; |A0A087WUU9|	RHSAS(0.59)NLHALAHPAPSPGSCSPK
-4.49	0.00555	TRA2B; Transformer-2 protein homolog beta|P62995|	S(1)RS(0.99)YSRDYR
-4.48	2.74E-05	MINT; Msx2-interacting protein |Q96T58|	DLEPGEVPS(1)DS(1)DEDGEHK
-4.47	0.00072	PSIP1; PC4 and SFRS1-interacting protein|O75475|	S(1)LAEQRQHEEANK
-4.45	4.53E-05	NOLC1; Nucleolar and coiled-body phosphoprotein 1 |Q14978|	S(1)PAVKPAAAPK
-4.41	0.00040	MKI67; Proliferation marker protein Ki-67|P46013|	LDQPGNLPGS(1)NRR
-4.32	0.00064	CBX5; Chromobox protein homolog 5|P45973|	S(1)NFSNSADDIK
-4.30	0.00140	KPNA2; Importin subunit alpha-1|P52292|	STNENANT(1)PAAR
-4.29	0.00082	TRA2A; Transformer-2 protein homolog alpha |Q13595|	S(1)RS(1)HS(1)PMSNR
-4.27	0.00045	GOLGA2; Golgin subfamily A member 2|A0A0C4DGS5|	LREYQQRNS(0.99)PGVPTGAK
-4.25	8.83E-05	MINT; Msx2-interacting protein |Q96T58|	DLEPGEVPS(1)DS(1)DEDGEHK
-4.22	0.00946	NCL; Nucleolin|P19338|	VAVAT(1)PAKK
-4.18	0.00144	MKI67; Proliferation marker protein Ki-67|P46013|	RRS(0.97)GASEANLIVAK
-4.13	0.00015	RPA1; Replication protein A 70 kDa DNA-binding subunit |P27694|	GARVS(1)DFGGR
-4.11	0.01401	TOR1AIP1; Torsin-1A-interacting protein 1 |A0A0A0MSK5|	RGLRDS(1)HS(1)S(1)EEDEASSQTDLSQTI
-4.10	0.00029	SMTN; Smoothelin|P53814|	VNS(1)PGTLAR
-4.07	0.00325	RFX7; DNA-binding protein RFX7|H0YLX2|	HHDTHFGRLT(0.99)PVS(1)PVQHQGAT
-4.04	1.98E-05	PPP1R14B; Protein phosphatase 1 regulatory subunit 14B|Q96C90|	VYFQS(1)PPGAAGEGPGGADDEGPVRR
-4.04	0.00123	DIDO1; Death-inducer obliterator 1 |Q9BTC0|	SILAKPS(0.70)SSPDPR
-3.99	0.01379	ATXN2L; Ataxin-2-like protein|Q8WWM7|	TLSS(0.88)PSNRPSGETSVPPPPAVGR
-3.99	0.00070	TRA2A; Transformer-2 protein homolog alpha|Q13595|	S(1)RS(1)HS(1)PMSNR
-3.98	0.00013	VAPB; Vesicle-associated membrane protein-associated protein B/C |O95292|	IISTTASKTET(0.98)PIVSK
-3.95	3.97E-05	LAD1; Ladinin-1 |O00515|	RATASEQPLAQEPPASGGS(0.98)PATTK
-3.94	0.00039	CLCC1; Chloride channel CLIC-like protein 1|Q96S66|	FQTGNKS(1)PEVLR
-3.94	4.79E-05	MAZ; Myc-associated zinc finger protein |I3L2Z5|	QVHS(0.86)TERPFK
-3.91	0.00326	TRA2A; Transformer-2 protein homolog alpha |Q13595|	S(1)RS(1)HS(1)PMSNR
-3.89	0.0002	MDC1; Mediator of DNA damage checkpoint protein 1|Q14676|	NRS(0.81)SGKT(1)PETLVPTAPK
-3.88	0.00193	UTP18; U3 small nucleolar RNA-associated protein 18 homolog|Q9Y5J1|	VQEHEDS(1)GDS(1)EVENEAK
-3.88	0.00240	METAP2; Methionine aminopeptidase 2|P50579|	QLERS(1)ALEDK
-3.87	0.00494	AATF; Protein AATF|Q9NY61|	YLVDGTKPNAGS(0.99)EEIS(1)S(1)EDDE
-3.86	0.00021	PTPN1; Tyrosine-protein phosphatase non-receptor type 1|P18031|	VVGGSLRGAQAAS(1)PAK

Supplementary Table S4

-3.86	0.00113	GPATCH2; G patch domain-containing protein 2 |Q9NW75|	KDHS(1)DS(1)DDQMLVAK
-3.86	0.00069	NEDD1; Protein NEDD1|Q8NHV4|	LVTSGAESGNLNTS(0.99)PSSNQTR
-3.85	0.00205	NUP98; Nuclear pore complex protein Nup98-Nup96 |P52948|	ALTT(0.95)PTHYKLT(0.87)PRPATR
-3.85	0.00107	TMPO; Lamina-associated polypeptide 2, isoforms beta/gamma |P42167|	HAS(1)PILPITEFSDIPR
-3.85	0.00632	MCM4; DNA replication licensing factor MCM4 |P33991|	QRPDLGS(1)AQK
-3.77	0.00216	MKI67; Proliferation marker protein Ki-67|P46013|	TFKES(1)PK
-3.76	0.00014	DDX20; Probable ATP-dependent RNA helicase DDX20|Q9UHI6|	S(0.99)YLEGSSDNQLK
-3.76	8.15E-05	NUMA1; Nuclear mitotic apparatus protein 1|Q14980|	LGS(1)PDYGNSALLSLPGYRPTTR
-3.71	0.00193	GTSE1; G2 and S phase-expressed protein 1|Q9NYZ3|	LLASS(0.92)PALPSSGAQAR
-3.66	0.00015	MAP7D1; MAP7 domain-containing protein 1|Q3KQU3|	WSWAGALHHS(0.61)SPGHK
-3.64	2.58E-05	PDLIM1: PDZ and LIM domain protein 1|O00151|	SAMPFTAS(0.99)PASSTTAR
-3.61	0.00055	NCKAP5L; Nck-associated protein 5-like|H7C1V4|	VPTSAPSLGKPNKS(0.93)PHS(0.66)SPTK
-3.60	0.01068	MTDH; Protein LYRIC|Q86UE4|	WNSVS(0.99)PASAGK
-3.59	0.00123	AATF; Protein AATF|Q9NY61|	DGTKPNAGS(0.99)EEIS(1)S(1)EDDELVE
-3.59	0.00125	KLC3; Kinesin light chain 3|Q6P597|	TLSASTQDLS(1)PH
-3.58	0.00812	MYO9B; Unconventional myosin-IXb|M0R300|	KKPGDASSLPDAGLS(0.97)PGSQVDSK
-3.58	0.00378	DDX39A; ATP-dependent RNA helicase DDX39A |O00148|	DLLDYDEEEEPQAPQES(0.59)TPAPPKK
-3.54	1.66E-05	MKI67; Proliferation marker protein Ki-67|P46013|	ELFQT(1)PVCTDKPTTHEK
-3.53	0.00221	NUP98; Nuclear pore complex protein Nup98-Nup96|P52948|	ALTT(0.89)PTHYKLT(0.98)PRPATR
-3.51	0.01230	MYBBP1A; Myb-binding protein 1A |Q9BQG0|	SPAPGAPTRS(0.87)PSTPAK
-3.50	0.00016	OSBPL11; Oxysterol-binding protein-related protein 11|Q9BXB4|	VSESEGKLEGQATAVT(1)PNK
-3.49	0.00079	DDX39A; ATP-dependent RNA helicase DDX39A|O00148|	DLLDYDEEEEPQAPQEST(0.74)PAPPKK
-3.49	0.00057	RFX7; DNA-binding protein RFX7|H0YLX2|	HHDTHFGRLT(0.99)PVS(1)PVQHQGAT
-3.45	0.01152	EPS8; Epidermal growth factor receptor kinase substrate 8|Q12929|	QNS(0.93)SSSDSGGSIVR
-3.34	0.00435	NONO; Non-POU domain-containing octamer-binding protein|Q15233|	FGQAATMEGIGAIGGT(1)PPAFNR
-3.32	0.00024	AHNAK; Neuroblast differentiation-associated protein AHNAK|Q09666|	LSGPS(1)LK
-3.28	0.00035	NCAPD2; Condensin complex subunit 1|Q15021|	YQPLASTASDNDFVT(1)PEPR
-3.25	0.00279	ACIN1; Apoptotic chromatin condensation inducer in the nucleus|E7EQT4|	S(1)KS(1)PS(0.98)PPRLTEDR
-3.24	0.00155	CLASP1; CLIP-associating protein |Q7Z460|	NSSNTSVGS(0.94)PSNTIGRTPSR
-3.22	0.00225	RTN4; Reticulon-4 |Q9NQC3|	RRGS(0.73)SGS(0.78)VDETLFALPAASEP
-3.22	0.00400	PPP1R10; Serine/threonine-protein phosphatase 1 regulatory subunit 10 |Q96QC0|	RQSNVAAPGDAT(1)PPAEK
-3.20	0.00017	TJP2; Tight junction protein ZO-2|Q9UDY2|	LISDFEDTDGEGGAYT(1)DNELDEPAEEPL
-3.16	0.00132	GPATCH2; G patch domain-containing protein 2 |Q9NW75|	KDHS(1)DS(1)DDQMLVAK
-3.15	0.01275	EXOSC10; Exosome component 10 |Q01780|	VSSQFDPNKQT(0.80)PSGKK
-3.15	7.68E-06	NCKAP5L; Nck-associated protein 5-like |H7C1V4|	VPTSAPSLGKPNKS(0.93)PHS(0.66)SPTK
-3.12	0.00130	CHMP4B; Charged multivesicular body protein 4b |Q9H444|	NLLEIS(0.99)GPETVPLPNVPSIALPSKPA
-3.10	0.00416	DYNC1LI1; Cytoplasmic dynein 1 light intermediate chain 1|Q9Y6G9|	SVSSNVASVS(0.96)PIPAGSK
-3.10	0.00016	DLGAP5; Disks large-associated protein 5|Q15398|	NTASQNS(1)ILEEGETK
-3.08	0.00122	HMGA1; High mobility group protein HMG-I/HMG-Y |P17096|	KQPPVS(1)PGTALVGSQK
-3.07	0.00234	MKI67; Proliferation marker protein Ki-67|P46013|	DINTFLGT(1)PVQK
-3.06	0.00382	MYBBP1A; Myb-binding protein 1A|Q9BQG0|	PGAPTRSPS(0.54)T(0.54)PAKS(0.99)PK
-3.04	0.00088	MAP7D1; MAP7 domain-containing protein 1|Q3KQU3|	EEAKES(0.99)PSAAGPEDK
-3.02	0.00057	PPP1R10; Serine/threonine-protein phosphatase 1 regulatory subunit 10|Q96QC0|	INVQEILTSIMGS(0.97)PNSHPSEELLK
-2.99	0.01269	TPX2; Targeting protein for Xklp2|Q9ULW0|	S(1)PAFALK
-2.95	0.00360	SLC4A7; Anion exchange protein |C9JRP1|	KHS(1)DPHLLER
-2.93	0.00251	ADRM1; Proteasomal ubiquitin receptor ADRM1 |Q16186|	SQSAAVT(0.99)PSSTTSSTR
-2.91	0.00057	MYBBP1A; Myb-binding protein 1A|Q9BQG0|	S(1)PLSALAR
-2.88	0.00028	PCM1; Pericentriolar material 1 protein|Q15154|	LRDQHLNNS(0.96)SSSPQRSVDQR
-2.86	0.01086	DDX21; Nucleolar RNA helicase 2 |Q9NR30|	DCNPSEAASEESNS(0.67)EIEQEIPVEQK
-2.84	0.00298	LMNB2; Lamin-B2|Q03252|	AGGPAT(1)PLS(0.63)PTRLSR
-2.84	0.00579	ANKRD2; Ankyrin repeat domain-containing protein 2|Q9GZV1|	AEHNGLEGPNDS(0.91)GRETPQPVPAQ
-2.81	0.00492	RAB8A; Ras-related protein Rab-8A|P61006|	KLEGNS(1)PQGS(1)NQGVK
-2.79	0.00950	NIFK; MKI67 FHA domain-interacting nucleolar phosphoprotein|Q9BYG3|	EEIQETQT(0.99)PTHSR
-2.78	0.00281	C8orf33; UPF0488 protein C8orf33|Q9H7E9|	AALGHLAGEAAAAPGPGT(0.98)PCASR
-2.78	0.00243	TP53BP1; TP53-binding protein 1|Q12888|	IDEDGENTQIEDTEPMS(0.99)PVLNSK
-2.77	0.00011	MCM4; DNA replication licensing factor MCM4|P33991|	GLQVDLQS(1)DGAAAEDIVASEQSLGQK
-2.77	0.01547	SYVN1; E3 ubiquitin-protein ligase synoviolin|Q86TM6|	LES(1)PVAH
-2.76	3.6E-08	MKI67; Proliferation marker protein Ki-67|P46013|	KPVGEVHSQFSTGHANS(0.98)PCTIIIGK

Supplementary Table S4

-2.76	0.00028	SRRM2; Serine/arginine repetitive matrix protein 2|Q9UQ35|	DVNPGGKEET(0.93)PGQRPAVTETHQL
-2.75	0.00171	CHEK1; Serine/threonine-protein kinase Chk1|J3KN87|	VTSGGVSES(0.93)PSGFSK
-2.74	0.00062	NACC1; Nucleus accumbens-associated protein 1|Q96RE7|	RLWDS(1)GQK
-2.73	0.00342	SIPA1; Signal-induced proliferation-associated protein 1|F6RY50|	SVPSADSETPLTQDRPGS(0.84)PSGSEDK
-2.73	0.01251	ZNF644; Zinc finger protein 644 |Q9H582|	HVVS(1)PEQIATSDK
-2.70	0.00770	NOLC1; Nucleolar and coiled-body phosphoprotein 1 |Q14978|	LQT(1)PNT(1)FPK
-2.68	0.00051	PALB2; Partner and localizer of BRCA2 |Q86YC2|	QFDSSGS(0.92)PAKPHTTLQVSGR
-2.66	0.00112	ADAT1; tRNA-specific adenosine deaminase 1|Q9BUB4|	IAPGS(1)AK
-2.60	0.00381	ELAVL1; ELAV-like protein 1 |Q15717|	NVALLSQLYHS(1)PAR
-2.60	0.00599	PML; Protein PML|P29590|	AVS(1)PPHLDGPPS(1)PRS(1)PVIGSEVFL
-2.58	0.00749	KAT7; Histone acetyltransferase KAT7 |O95251|	NTADHDES(0.83)PPRT(0.89)PTGNAPSS
-2.56	0.00658	HNRNPA1; Heterogeneous nuclear ribonucleoprotein A1|P09651|	GGNFGGRSS(0.91)GPYGGGGQYFAK
-2.53	0.00076	CCD86; Coiled-coil domain-containing protein 86|Q9H6F5|	RALVEFESNPEET(0.58)REPGSPPSVQR
-2.52	3.28E-05	JPT1; Jupiter microtubule associated homolog 1 |Q9UK76|	MASNIFGT(1)PEENQASWAK
-2.51	0.00098	STAU2; Double-stranded RNA-binding protein Staufen homolog 2|A0A0A0MTD1|	GSSPT(0.98)PPCS(1)PVQPSK
-2.46	0.00083	ARHGEF7; Rho guanine nucleotide exchange factor 7|B1ALK7|	SGTLKS(1)PPK
-2.45	0.00225	MKI67; Proliferation marker protein Ki-67|P46013|	LDLLGNLPGS(1)K
-2.43	0.00950	JPT2; Jupiter microtubule associated homolog 2|Q9H910|	AMKPPGGESSNLFGS(0.99)PEEATPSSRP
-2.42	0.00078	DEK; Protein DEK|P35659|	S(1)LIVEGKREK
-2.41	0.00914	TJAP1; Tight junction-associated protein 1 |Q5JTD0|	HLHSGQEAAS(0.97)PGPAPSLAPGAVVP
-2.39	0.00252	NOLC1; Nucleolar and coiled-body phosphoprotein 1|Q14978|	LQT(1)PNT(1)FPK
-2.39	0.00871	TCOF; Treacle protein |Q13428|	VGDVT(1)PQVK
-2.38	0.00698	PPP1R2; Protein phosphatase inhibitor 2|P41236|	GDDEDACS(0.94)DTEATEAMAPDILARK
-2.38	0.00860	CDC42EP1; Cdc42 effector protein 1|Q00587|	ASWES(1)LDEEWRAPQAGSR
-2.37	0.00476	NUP153; Nuclear pore complex protein Nup153|P49790|	FSS(1)PIVK
-2.36	0.00116	MK67I; MKI67 FHA domain-interacting nucleolar phosphoprotein|Q9BYG3|	TVDS(0.99)QGPT(1)PVCT(0.97)PTFLER
-2.34	0.00951	LMNB2; Lamin-B2|Q03252|	AGGPATPLS(0.98)PTRLSR
-2.33	0.00715	SFPQ; Splicing factor, proline- and glutamine-rich|P23246|	GMGPGT(1)PAGYGR
-2.33	0.01197	RPRD2; Regulation of nuclear pre-mRNA domain-containing protein 2|Q5VT52|	QYPDS(0.99)PHPVPHR
-2.32	0.00039	NAP1L4; Nucleosome assembly protein 1-like 4|Q99733|	LDNVPHT(0.93)PSSYIETLPK
-2.29	9.88E-05	MEPCE; 7SK snRNA methylphosphate capping enzyme|Q7L2J0|	GGGPQAQSHGEARLS(1)DPPGR
-2.28	0.00214	KAT7; Histone acetyltransferase KAT7|O95251|	NTADHDES(0.80)PPRT(0.97)PTGNAPSS
-2.27	0.00503	HMMR; Hyaluronan mediated motility receptor |O75330|	RFNDPSGCAPS(0.99)PGAYDVK
-2.26	0.00034	HMGA1; High mobility group protein HMG-I/HMG-Y|P17096|	EPSEVPT(1)PK
-2.25	0.00180	CBX8; Chromobox protein homolog 8|Q9HC52|	RQDSDLVQCGVTS(0.76)PSSAEATGK
-2.24	0.00384	TOR1AIP1; Torsin-1A-interacting protein 1|A0A0A0MSK5|	VNFSEEGET(1)EEDDQDSSHSSVTTVK
-2.23	0.01114	HUWE1; E3 ubiquitin-protein ligase HUWE1 |Q7Z6Z7|	AGSST(0.82)PGDAPPAVAEVQGR
-2.22	0.00550	TNS3; Tensin-3|Q68CZ2|	LRKLS(1)LGQYDNDAGGQLPFSK
-2.21	0.01007	EAPP; E2F-associated phosphoprotein|G3V2W9|	VATAPTRYYDDIYFDS(1)DS(1)EDEDRAV
-2.19	0.00763	MAP4; Microtubule-associated protein|E7EVA0|	DGVLTLANNVT(1)PAK
-2.15	0.00402	WNK2; Serine/threonine-protein kinase WNK2|F8W9F9|	RPEQQDVSS(0.94)PAK
-2.15	0.00165	NAP1L4; Nucleosome assembly protein 1-like 4 |Q99733|	ADHS(1)FS(1)DGVPSDSVEAAK
-2.15	0.00197	TMPO; Lamina-associated polypeptide 2, isoform alpha |P42166|	SHISDQS(1)PLSSK
-2.14	0.00128	APC; Adenomatous polyposis coli protein|P25054|	SGRS(0.82)PTGNTPPVIDSVSEK
-2.10	0.00299	CDCA3; Cell division cycle-associated protein 3 |Q99618|	LGRS(0.99)PLTILQDDNS(0.99)PGTLTLR
-2.10	0.00096	PARG; Poly(ADP-ribose) glycohydrolase|Q86W56|	WGAATTS(0.95)PAASDAR
-2.07	0.00245	CDCA3; Cell division cycle-associated protein 3|Q99618|	LGRS(0.99)PLTILQDDNS(0.99)PGTLTLR
-2.07	0.00604	MKI67; Proliferation marker protein Ki-67|P46013|	ELFQT(1)PDHTEESTTDDK
-2.07	0.00895	PPP1R18; Phostensin|A0A0G2JHC2|	DWRQS(1)PGEVGDR
-2.06	0.01277	MDC1; Mediator of DNA damage checkpoint protein 1|Q14676|	TVPIAPELPPSTSTEQPVT(0.71)PEPTSR
-2.05	0.00319	MRTFB; Myocardin-related transcription factor B |Q9ULH7|	SGEISLPIKEEPS(1)PISK
-2.04	0.00844	A0A087WUL7;|A0A087WUL7|	DAAAHLQTSHKPSAEDAEGQS(1)PLSQK
-2.04	0.00069	ANLN; Anillin |Q9NQW6|	SCEGQNPELLPKT(1)PIS(1)PLK
-2.01	0.00014	RRM2; Ribonucleoside-diphosphate reductase subunit M2 |P31350|	ENT(1)PPALSGTR
-2.01	0.01083	DIDO1; Death-inducer obliterator 1|Q9BTC0|	STT(0.71)PPGS(0.99)PPPPPLPEPPVLK
-2.01	0.00952	MKI67; Proliferation marker protein Ki-67|P46013|	ELFQT(1)PGHTEELVAAGK
-2.00	0.00044	PHF14; PHD finger protein 14|O94880|	SNEDS(1)LILEK
-1.99	0.00416	PDLIM5; PDZ and LIM domain protein 5|Q96HC4|	EVVKPVPITS(0.99)PAVSK

Supplementary Table S4

-1.95	0.00180	RBM15B; Putative RNA-binding protein 15B|Q8NDT2|	TTHS(0.99)PYEER
-1.95	0.00103	RACGAP1; Rac GTPase-activating protein 1|Q9H0H5|	VSLLGPVTT(0.80)PEHQLLK
-1.95	0.00469	PBK; Lymphokine-activated killer T-cell-originated protein kinase|Q96KB5|	GLSHS(1)PWAVK
-1.95	9.87E-06	RICTROR; Rapamycin-insensitive companion of mTOR|Q6R327|	HIEDTGS(0.90)TPS(0.98)IGENDLK
-1.94	0.00252	MDC1; Mediator of DNA damage checkpoint protein 1|Q14676|	DLEPFTPTDQSVT(0.93)PEAIAQGGQSK
-1.92	0.00451	NECAP2; Adaptin ear-binding coat-associated protein 2|Q9NVZ3|	VRPAS(0.74)TGGLSLLPPPPGGK
-1.90	0.00869	NUP107; Nuclear pore complex protein Nup107|P57740|	RVLLQASQDENFGNTT(0.78)PRNQVIPR
-1.90	0.00025	TNKS1BP1; 182 kDa tankyrase-1-binding protein|Q9C0C2|	VNLFPGLS(0.87)PSALK
-1.88	0.00762	PDLIM1; PDZ and LIM domain protein 1|O00151|	VWS(1)PLVTEEGK
-1.87	0.00786	TP53BP1; TP53-binding protein 1|Q12888|	TGTRETAVPGPLGIEDIS(1)PNLS(1)PDDK
-1.87	0.00968	KI67; Proliferation marker protein Ki-67|P46013|	ELFQT(0.99)PGTDKPTTDEK
-1.87	0.00798	CASC3; Protein CASC3|O15234|	LEQTSVRDPS(0.97)PEADAPVLGS(1)PEK
-1.86	0.00088	ANKHD1; Ankyrin repeat and KH domain-containing protein 1|Q8IWZ3|	TVSLPLSS(0.99)PNIK
-1.84	0.00685	ANKZF1; Ankyrin repeat and zinc finger domain-containing protein 1 |Q9H8Y5|	LAAQLGAPTS(0.92)PIPDSAIVNTRR
-1.83	0.00040	LAMTOR1; Ragulator complex protein LAMTOR1|Q6IAA8|	LLLDPSS(0.97)PPTK
-1.83	0.00195	RBMXL1; RNA binding motif protein, X-linked-like-1|Q96E39|	GLPPS(1)VER
-1.82	0.00145	PCBP1; Poly(rC)-binding protein 1|Q15365|	DLEGPPLDAYSIQGQHT)IS(0.88)PLDLAK
-1.82	0.00394	CTTN; Src substrate cortactin|Q14247|	DY(1)SSGFGGK
-1.81	0.00255	MARK2; Serine/threonine-protein kinase MARK2 |Q7KZI7|	EGDTITLKPRPSADLTNSSAPS(0.89)PSHK
-1.80	0.00057	ATF7IP; Activating transcription factor 7-interacting protein 1|Q6VMQ6|	EAFLVLS(1)DEEDISGEK
-1.78	0.00835	KPNA3; Importin subunit alpha-4|O00505|	KRNVPQEES(1)LEDS(1)DVDADFK
-1.76	0.00554	NCBP3; Nuclear cap-binding protein subunit 3|Q53F19|	MIST(0.98)PS(0.99)PKK
-1.76	0.00683	HDLBP; High density lipoprotein binding protein (Vigilin), isoform CRA_a|A0A024R4E5|	DCDPGS(1)PRRCDIIIISGR
-1.72	0.00529	ZYX; Zyxin|Q15942|	FTPVASKFS(1)PGAPGGSGSQPNQK
-1.71	0.00092	MYBBP1A; Myb-binding protein 1A|Q9BQG0|	LSLVIRS(0.81)PSLLQSGAK
-1.70	0.00136	CIC; Protein capicua homolog|I3L2J0|	SRQPS(0.77)PLLLLPPPAGLTSDPGPSVR
-1.70	0.00039	CASP8AP2; CASP8-associated protein 2|A0A087WTW5|	VLRNES(1)PPQVPVYNNSHK
-1.69	0.00945	RRBP1; Ribosome-binding protein 1|A0A0A0MRV0|	NTDVAQS(1)PEAPKQEAPAKK
-1.68	0.00238	TOP2A; DNA topoisomerase 2-alpha|P11388|	MEVS(1)PLQPVNENMQVNK
-1.66	0.00950	RRP1B; Ribosomal RNA processing protein 1 homolog B |Q14684|	PLHGVLKT(0.88)PT(0.81)SSPASSPLVAK
-1.61	0.00066	SHC1; SHC-transforming protein 1|P29353|	ELFDDPSY(0.74)VNVQNLDK
-1.61	0.01055	MAP7; Ensconsin |Q14244|	LFVT(0.99)PPEGSSR
-1.59	0.00645	FAM83H; Protein FAM83H|Q6ZRV2|	GS(0.99)PTSAYPERK
-1.58	0.00768	BCLFA1; Bcl-2-associated transcription factor 1|Q9NYF8|	DTFEHDPSES(1)IDEFNK
-1.58	0.00069	RGPD3; RanBP2-like and GRIP domain-containing protein 3|A6NKT7|. RGPD1; RanBP2-like and GRIP domain-containing protein|P0DJD0|. RANBP2; E3 SUMO-protein ligase RanBP2|P49792|. RGPD4; RanBP2-like and GRIP domain-containing protein 4 |Q7Z3J3|. RGPD5; RanBP2-like and GRIP domain-containing protein 4|Q99666|	ITPHVS(0.63)RS(0.71)S(0.71)T(0.69)PR
-1.57	0.00024	CAVIN1; Caveolae-associated protein 1 |Q6NZI2|	SFT(1)PDHVVYAR
-1.56	0.00373	CDC42EP1; Cdc42 effector protein 1|Q00587|	QARAS(1)WES(0.99)LDEEWRAPQAGSR
-1.56	0.00010	ARFGAP1; ADP-ribosylation factor GTPase-activating protein 1 |Q8N6T3|	LAEGREWSLESSPAQNWT(0.98)PPQPR
-1.51	0.00591	DCP2; m7GpppN-mRNA hydrolase |H0Y9T5|	FRHSQQLFPDGS(1)PGDQWVK
-1.50	0.00530	SCRIB; Protein scribble homolog |A0A0G2JNZ2|	ALS(1)PAELR
-1.46	0.00639	PGAM1; Phosphoglycerate mutase 1|P18669|	S(0.99)YDVPPPPMEPDHPFYSNISK
-1.42	0.00864	TMCC3; Transmembrane and coiled-coil domain protein 3|Q9ULS5|	RGGS(0.68)DTNLNFDVPDGILDFHK
-1.41	0.00430	UCHL3; Ubiquitin carboxyl-terminal hydrolase isozyme L3|P15374|	FLEESVSMS(0.99)PEERAR
-1.37	0.00749	MAPK1IP1L; MAPK-interacting and spindle-stabilizing protein-like |Q8NDC0|	SDEFSLADALPEHS(1)PAK
-1.36	0.00279	NUCKS1; Nuclear ubiquitous casein and cyclin-dependent kinase substrate 1|Q9H1E3|	ATVT(1)PS(1)PVK
-1.36	0.00791	TOP2A; DNA topoisomerase 2-alpha|P11388|	TQMAEVLPS(1)PR
-1.36	0.00422	JPT2; Jupiter microtubule associated homolog 2 |Q9H910|	TSDIFGS(1)PVTATSR
-1.33	0.00152	ZC3HAV1; Zinc finger CCCH-type antiviral protein 1 |C9J6P4|	GESTAPAQVSTLPQS(0.91)PAALSSSNR
-1.27	0.00634	MDC1; Mediator of DNA damage checkpoint protein 1|Q14676|	EDS(1)DT(1)DVDDDSRPPGRPAEVHLER
-1.24	0.00118	RAB1A; Ras-related protein Rab-1A|P62820|	IQST(0.81)PVK
-1.23	0.00361	ANLN; Anillin|Q9NQW6|	GNIWS(1)AEK
-1.22	0.00737	NFIA; Nuclear factor 1 |B1AKN5|	SGFSSPS(0.97)PSQTSSLGTAFTQHHR
-1.19	0.00036	DBNL; Drebrin-like protein|Q9UJU6|	YQEQGGEAS(0.99)PQRTWEQQQEVVSR
-1.17	0.00672	RAVER1; Ribonucleoprotein PTB-binding 1|A0A087WZ13|	AIGSS(0.84)PLGSGEGLLGLS(1)PGPNGHSHLLK
-1.13	0.00350	HAUS6; HAUS augmin-like complex subunit 6|Q7Z4H7|	NQIPRT(1)PENLITEIR
-1.08	0.00061	TPR; Nucleoprotein TPR|P12270|	EGVQGPLNVS(0.96)LSEEGK
-1.04	0.00399	AHNAK; Neuroblast differentiation-associated protein AHNAK|Q09666|	SRVDIETPNLEGTLTGPRLGS(0.99)PSGK

-1.03	0.00182	SRRM2; Serine/arginine repetitive matrix protein 2|Q9UQ35|	QGSITS(0.7)PQANEQSVT(0.94)PQRR
-1.02	0.00250	DKC1; H/ACA ribonucleoprotein complex subunit DKC1|O60832|	S(1)LPEEDVAEIQHAEEFLIKPESK
-1.01	0.00496	CHD3; Chromodomain-helicase-DNA-binding protein 3|H7C3H7|	GRPPAQALGPAAS(1)PPPS(1)PPLGPSLG
-1.01	0.00048	CRYBG1; Beta/gamma crystallin domain-containing protein 1 |A0A0J9YWL0|	DTCVQS(1)PISSFPCTDLK
-0.817	0.00246	GATAD2A; Transcriptional repressor p66-alpha|Q86YP4|	TSAQANSTPTSVASVVTSAES(0.99)PASR
-0.70	0.00021	RAD18; E3 ubiquitin-protein ligase RAD18|Q9NS91|	NHLLQFALES(1)PAKS(0.99)PASSSSK

Supplementary Table S4. Significantly decreased phosphopeptide relative abundance level in A431/CCKBR cells after EBRT. MS2-based quantification shown as log2 ratio, *P*<0.05.

LOG$_2$ RATIO: ^{177}Lu-PP-F11N/CON	*P*-value	UniProt	PROTEIN
5.27	2.6E-06	A0A0C4DH73	IGKV1-12; Protein IGKV1-12
4.44	2.5E-05	Q9BQ67	GRWD1; Glutamate-rich WD repeat-containing protein 1
3.37	0.0001	P52594	AGFG1; Arf-GAP domain and FG repeat-containing protein 1
2.47	3.2E-05	O43815	STRN; Striatin
2.41	2.6E-05	Q7Z4H3	HDDC2; HD domain-containing protein 2
2.25	0.001	Q13751	LAMB3; Laminin subunit beta-3
2.16	9.5E-05	P53539	FOSB; Protein fosB
1.82	0.0001	P53814	SMTN; Smoothelin
1.65	6.3E-05	H3BRB3	KIF22; Kinesin-like protein
1.64	0.0005	Q15750	TAB1; TGF-beta-activated kinase 1 and MAP3K7-binding protein 1
1.62	0.0002	O96005	CLPT1; Cleft lip and palate transmembrane protein
1.46	0.0001	I3VM54	KDM2A; Lysine-specific demethylase 2A
1.09	0.0010	Q7Z4H7	HAUS6; HAUS augmin-like complex subunit 6
1.01	0.0003	O00622	CYR61; Protein CYR61
1.00	0.0141	Q86YV9	HPS6; Hermansky-Pudlak syndrome 6 protein
0.94	0.0118	Q5QP56	BCL2L1; Bcl-2-like protein 1
0.86	0.0121	Q02952	AKA12; A-kinase anchor protein 12
0.84	0.0077	Q02040	AK17A: A-kinase anchor protein 17A
0.821	0.0010	S4R3V8	LSR; Lipolysis-stimulated lipoprotein receptor
-1.46	0.0002	Q15018	FAM175B; BRISC complex subunit Abro1
-1.55	0.0065	Q92667	AKAP1; A-kinase anchor protein 1, mitochondrial
-1.60	0.0002	Q9Y6B6	SAR1B; GTP-binding protein SAR1b
-1.97	6.0E-05	Q5HYI8	RABL3; Rab-like protein 3
-2.55	0.0005	P35580	MYH10; Myosin-10
-2.60	0.0002	Q8WUY1	THEM6; Protein THEM6

Supplementary Table S5. Significant changes in relative total protein abundance levels after treatment with [^{177}Lu]Lu-PP-F11N in A431/CCKBR cell. MS2-based quantification shown as log2 ratio, P<0.05

Supplementary Table S6

LOG$_2$ RATIO: EBRT/CON	*P*-value	UniProt	PROTEIN
1.55	0.0032	Q15392	DHCR24; Delta(24)-sterol reductase
1.39	0.0019	H7BZV4	ARL6IP4; ADP-ribosylation factor-like protein 6-interacting protein 4
1.32	0.0131	P23786	CPT2; Carnitine O-palmitoyltransferase 2, mitochondrial
0.80	0.0069	E9PL01	SPCS2; Signal peptidase complex subunit 2
0.69	0.0017	P14927	UQCRB; Cytochrome b-c1 complex subunit 7
0.68	0.0014	Q9NRN7	AASDHPPT;
0.60	0.0023	Q8IZP0	ABI1; Abl interactor 1
-0.87027	0.0091	Q14165	MLEC; Malectin
-0.89371	0.0007	Q9BVL2	NUP58; Nucleoporin p58/p45
-1.15661	0.0062	Q7Z4H3	HDDC2; HD domain-containing protein 2
-1.79024	0.0001	Q9UPN7	PPP6R1; Serine/threonine-protein phosphatase 6 regulatory subunit 1
-1.7997	0.0036	Q92882	OSTF1; Osteoclast-stimulating factor 1
-1.83217	0.0003	Q8N983	MRPL43; 39S ribosomal protein L43, mitochondrial
-1.90071	0.0024	Q86W92	PPFIBP1; Liprin-beta-1
-2.06716	0.0008	P16435	POR; NADPH--cytochrome P450 reductase

Supplementary Table S6. Significant changes in relative total protein abundance levels after EBRT in A431/CCKBR cells. MS2-based quantification shown as log2 ratio, $P<0.05$.

Supplementary Table S7. Comparative analysis of cellular response to TRT with [^{177}Lu]Lu-PP-F11N and EBRT. Bioinformatics analysis of the protein groups with altered expression or phosphorylation levels in response to PRRT or EBRT identified by proteomics and phosphoproteomics analysis. Fold Enrichments (Fold En.) for identified biological processes (GOTERM) or pathways (BIOCARTA, KEGG PATHWAYS) were scored for terms with *P*<0.01 in a group of at least 3 proteins.

TRT (^{177}Lu-PP-F11N)	*P-value*<br>Fold En.	EBRT	*P-value*<br>Fold En.
**DNA damage response, repair and nucleus structure**			
**Cellular response to DNA damage stimulus** GOTERM_BP (DDX39A, RAD18, YAP1DTL, MAPK1, TP53, UBA1, USP10)	*5.0E-3*<br>3.8	**Cellular response to DNA damage stimulus** GOTERM_BP (APC, DDX39A, RAD18, RAD50, YAP1, AATF, CHEK1, CHEK2, RNF168, TOP2A, TP53BP1, TP53)	*3.5E-4*<br>3.8
**DNA damage response, signal transduction by p53 class mediator** GOTERM_BP (FOXO3, TP53, USP10)	*9.6E-3*<br>19.9	**DNA damage response, signal transduction by p53 class mediator** GOTERM_BP (GTSE1, CHEK2, NPM1, PML, TNKS1BP1, TP53)	*2.5E-3*<br>6.3
**DNA damage response, detection of DNA damage** GOTERM_BP (RAD18, DTL, RFC1, RPA1)	*5.5E-3*<br>11.0	**Double-strand break repair via nonhomologous end joining** GOTERM_BP (RAD50, HIST3H3, MDC1, RIF1, RNF168, TP53BP1, UIMC1)	3.8E-4<br>7.3
**Translesion synthesis** GOTERM_BP (DTL, RFC1, RPA1, USP10)	*5.5E-3*<br>11.0	**Double-strand break repair via homologous recombination** GOTERM_BP (RAD50, RAD51AP1, NUCKS1, PALB2, RPA1, SFPQ)	*5.3E-3*<br>5.3
**Nucleosome assembly** - GOTERM_BP (HIST1H1B, HIST1H1D, HIST2H3A, MCM2, NPM1, SART3)	*6.9E-3*<br>5.0	**Base-excision repair** GOTERM_BP (HUWE1, HMGA1, NTHL1, RPA1, TP53)	*1.9E-3*<br>9.4
**Sister chromatid cohesion** GOTERM_BP (AHCTF1, RANBP2, CENPC, CENPF, KIF22, RPS27)	*3.8E-3*<br>5.8	**Interstrand cross-link repair** GOTERM_BP (RAD51AP1, NUCKS1, RPA1, RNF168, USP1)	*6.4E-3*<br>6.7
**Nuclear pore complex assembly** - GOTERM_BP (AHCTF1, NUP153, NUP93)	*5.2E-3*<br>27.1	**Nuclear pore complex assembly** - GOTERM_BP (NUP107, NUP153, NUP98, RTN4, TPR)	*1.6E-5*<br>29.8
		**DNA replication** GOTERM_BP (POLE, GINS2, RAD50, UPF1, CHEK1, RRM2, CHAF1A, DUT, KAT7, MCM2, MCM4, MCM6, NFIA, NFIC, NUP98, RPA1)	*1.4E-8*<br>6.8
**RNA processing, transport and metabolism**			
**mRNA splicing, via spliceosome** GOTERM_BP (DDX39A, POLR2A, FIP1L1, HNRNPH1, HNRNPU, RAVER1, SRRM1, SRRM2, SF3B2, SART3, TRA2A)	*8.1E-5*<br>4.9	**mRNA splicing, via spliceosome** GOTERM_BP (DDX39A, ELAVL1, POLR2A, SNW1, YBX1, CASC3, HNRNPA1, HNRNPM, NONO, PABPN1, PCBP1, RAVER1, SRRM1, SRRM2, SPEN, SYNCRIP, TRA2A, TRA2B)	*5.2E-8*<br>5.3
**mRNA export from nucleus** GOTERM_BP (AGFG1, FIP1L1, DDX39A, RANBP2, NUP153, NUP50, NUP93, SRRM1)	*6.5E-5*<br>7.9	**mRNA export from nucleus** GOTERM_BP (DDX39A, UPF1, CASC3, NUP107, NUP153, NUP58, NUP98, SRRM1, TPR)	*1.4E-4*<br>5.9
**tRNA export from nucleus** GOTERM_BP (RANBP2, NUP153, NUP50, NUP93)	*3.9E-3*<br>12.4	**tRNA export from nucleus** GOTERM_BP (NUP107, NUP153, NUP58, NUP98, TPR)	*1.3E-3*<br>10.2
**RNA transport** KEGG_PATHWAY (RANBP2, RGPD5, EIF4G1, NUP153, NUP50, NUP93, RPP30, SRRM1, TGS1)	*8.1E-4*<br>4.4	**mRNA processing** GOTERM_BP (ARL6IP4, DDX47, IWS1, KHSRP, RBM15B, RBMXL1, ADAR, ACIN1, CASC3, HNRNPA1, NONO, PRPF38B, SRRM1, SFPQ, THRAP3)	*6.2E-7*<br>5.5
**Gene silencing by RNA** GOTERM_BP (RANBP2, POLR2A, HIST2H3A, NUP153, NUP50, NUP93)	*5.2E-3*<br>5.4	**rRNA processing** GOTERM_BP (DDX47, DDX21, DIS3L2, UTP14A, UTP18, CCDC86, DKC1, EXOSC10, NOLC1, RRP1B, RPL29)	*1.6E-3*<br>3.4
**Nuclear-transcribed mRNA catabolic process, nonsense-mediated decay** GOTERM_BP (DCP1A, EIF4G1, RPL19, RPL3, RPS27, RPS6)	*6.9E-3*<br>5.0	**Negative regulation of transcription** GOTERM_BP (BCLAF1, GATAD2A, JUN, MYBBP1A, RB1, RBM15B, SNW1, SP100, ATF7IP, CBX3, CBX5, HMGA1, NONO, NACC1, PML, SPEN, SFPQ, TRIM28, TP53, UIMC1)	*2.3E-4*<br>2.6
		**RNA polymerase III transcription** KEGG_PATHWAY (ADAR, NONO, SFPQ)	*7.7E-3*<br>20.3
		**mRNA surveillance pathway** KEGG_PATHWAY (UPF1, ACIN1, CASC3, PABPN1, PPP2R5A, SRRM1)	*9.4E-3*<br>4.6
**Cell cycle regulation**			
**Mitotic nuclear division** GOTERM_BP (HAUS6, NSUN2, ARHGEF2, ANLN, CENPC, CENPF, KIF22, PPP1R12A, RPS6, SKA3, ZC3HC1)	*2.0E-4*<br>4.4	**Mitotic nuclear division** GOTERM_BP (DIS3L2, HAUS6, PBK, TPX2, ANLN, CDC26, CDCA3, DYNC1LI1, ENSA, NEDD1, NUMA1, NOLC1, TPR)	*4.2E-4*<br>3.4
**Mitotic nuclear envelope disassembly** GOTERM_BP (RANBP2, NUP153, NUP50, NUP93)	*9.6E-3*<br>9.0	**Mitotic nuclear envelope disassembly** GOTERM_BP (NUP107, NUP153, NUP58, NUP98, TPR	*4.4E-3*<br>7.5
**Mitotic cell cycle** GOTERM_BP (CENPC, CENPF, FAM175B, PAK2)	*7.9E-3*<br>9.7	**Cell cycle** GOTERM_BP (RB1, CDC26, CHEK1, CHEK2, MCM2, MCM4, MCM6, TP53)	*1.9E-3*<br>4.5
		**G2/M transition of mitotic cell cycle** - GOTERM_BP (HAUS6, RAB8A, TPX2, CCP110, CEP152, CHEK1, CHEK2, CLASP1, ENSA, HMMR, NEDD1, PCM1)	*7.8E-6*<br>5.7
		**G1/S transition of mitotic cell cycle** GOTERM_BP (POLE, RB1, MCM2, MCM4, MCM6, RPA1, RRM2)	*4.6E-3*<br>4.5
		**Mitotic cytokinesis** GOTERM_BP (APC, RACGAP1, ANLN, CHMP4B)	*9.4E-3*<br>9.0
**Signal transduction pathways**			
**TGF-beta receptor signaling pathway** - GOTERM_BP (FOS, JUN, TAB1, CGN, PXN, ZYX)	*2.3E-3*<br>6.5	**Ras protein signal transduction** GOTERM_BP (DHCR24, CRKL, JUN, RB1, SHC1, MAPK14, RREB1, TRIM28, TP53)	*1.1E-5*<br>8.4
**ErbB signaling pathway** KEGG_PATHWAY (ABL2, JUN, SOS1, EGFR, MAPK1, PAK2)	*3.3E-3*<br>5.9	**Regulation of signal transduction by p53 class mediator** GOTERM_BP (RAD50, TPX2, CHEK1, CHEK2, CHD3, CHD4, MTA2, MAPK14, PML, RPA1, TP53)	*2.0E-5*<br>5.8
**Angiotensin II mediated activation of JNK via Pyk2** BIOCARTA (JUN, SOS1,CALM1, EGFR, MAPK1)	*3.8E-3*<br>7.2	**ATM Signaling Pathway** - KEGG_PATHWAY (JUN, RAD50, CHEK1, CHEK2, TP53)	*2.6E-3*<br>8.1
**Signaling of Hepatocyte Growth Factor Receptor** BIOCARTA (FOS, JUN, SOS1, MAPK1, PXN)	*5.3E-3*<br>6.6	**Links between Pyk2 and Map Kinases** - KEGG_PATHWAY (CRKL, JUN, SHC1, MAPK14, MAP2K2)	*7.6E-3*<br>6.0
**Integrin Signaling Pathway** BIOCARTA (FOS, JUN, SOS1, MAPK1, PXN)	*5.8E-3*<br>6.5		
**Estrogen signaling pathway** - KEGG_PATHWAY (FOS, JUN, SOS1, CALM1, EGFR, MAPK1)	*5.7E-3*<br>5.1		
**TOR signaling** GOTERM_BP (LARP1, CCDC88A, RPS6)	*8.4E-3*<br>21.3		
**MAPK signaling pathway** - KEGG_PATHWAY (FOS, JUN, SOS1, TAB1, EGFR, MAPK1, PAK2, STMN1, TP53)	*8.8E-3*<br>3.0		
**Regulation of Rho protein signal transduction** GOTERM_ BP (FGD1, ARHGEF1, ARHGEF5, ARHGEF2, SOS1)	*8.9E-3*<br>6.1		
**Cell morphology, adhesion and phenotype**			
**Cell-cell adhesion** GOTERM_BP (BAG3, LIMA1, LARP1, SLK, ANLN, CGN, CTTN, EIF4G1, FAM129B, LAD1, MARK2, PAK2, PFKP, PPP1R13L, RSL1D1, TJP1, TJP2)	*1.4E-8*<br>6.2	**Cell-cell adhesion** GOTERM_BP (AHNAK, CDC42EP1, CRKL, LASP1, MKL2, NDRG1, PDLIM1, PDLIM5, PPFIBP1, RAB1A, ARHGEF16, VAPB, ABI1, ANLN, ATXN2L, CAST, CHMP4B, CTTN, DBNL, EIF4G1, GOLGA2, HDLBP, LAD1, MARK2, PCBP1, PTPN1, RTN4, RSL1D1, RPL29, TNKS1BP1, TMPO, TJP2, ZC3HAV1)	*1.4E-19*<br>8.0
**Regulation of actin cytoskeleton** KEGG_PATHWAY (FGD1, ARHGEF1, SOS1, EGFR, ITGB4, MAPK1, PAK2, PXN, PPP1R12A)	*2.9E-3*<br>3.6	**Actin cytoskeleton reorganization** GOTERM_BP (RICTOR, SHC1, CTTN, EPS8, PTPN1, SIPA1L1)	*7.1E-4*<br>8.4
**Regulation of cytoskeleton organization** GOTERM_BP (ARHGEF5, MARK2, MAPK1, STMN1)	*8.5E-4*<br>20.9		
**Focal adhesion** KEGG_PATHWAY (JUN, SOS1, EGFR, ITGB4, LAMB3, MAPK1, PAK2, PXN, PPP1R12A, ZYX)	*6.0E-4*<br>4.1		
**Tight junction** KEGG_PATHWAY (CGN, CLDN4, CTTN, MYH10, TJAP1, TJP1, TJP2)	*4.9E-4*<br>6.8		
**Cell proliferation** GOTERM_BP (BCL2L1, LARP1, ARHGEF1, YAP1, CENPF, CCKBR, CYR61, DKC1, EGFR, FAM83B, MYH10, RPS27, TP53)	*3.2E-4*<br>3.5		
**Transport and protein modifications**			
**Regulation of glucose transport** GOTERM_BP (RANBP2, NUP153, NUP50, NUP93)	*4.3E-3*<br>12.0	**Regulation of glucose transport** GOTERM_BP (NUP107, NUP153, NUP58, NUP98, TPR)	*1.5E-3*<br>9.9
**Protein sumoylation** GOTERM_BP (RANBP2, HDAC7, MDC1, NUP153, NUP50, NUP93, RPA1, TP53)	*1.7E-4*<br>6.8	**Protein sumoylation** GOTERM_BP (SP100, CBX8, MDC1, NUP107, NUP153, NUP58, NUP98, PML, RPA1, RNF168, TOP2A, TPR, TRIM28, TP53BP1, TP53)	*2.8E-9*<br>8.4
**Proteoglycans in cancer** KEGG_PATHWAY (ARHGEF1, SOS1, CTTN, EGFR, MAPK1, PXN, PPP1R12A, RPS6, TP53)	*2.1E-3*<br>3.8	**Protein import into nucleus** GOTERM_BP (ADAR, KPNA2, KPNA3, NUP107, NUP153, NUP58, PPP1R10, TPR)	*2.7E-5*<br>9.0
