# Supplementary material for: Comparative analysis of cancer cell responses to targeted radionuclide therapy (TRT) and external beam radiotherapy (EBRT)
Source: J Hematol Oncol. 2022 Aug 31;15:123. doi: 10.1186/s13045-022-01343-y (PMC9429584; doi:10.1186/s13045-022-01343-y)
Supplement: Supplementary file 3 — Additional file 3. Supplementary Methods. [file 13045_2022_1343_MOESM3_ESM.pdf]

**Supplementary Methods**

# Comparative analysis of cancer cell responses to targeted radionuclide therapy (TRT) and external beam radiotherapy (EBRT).

Michal Grzmil[1], Paul Boersema[2], Ashish Sharma[3], Alain Blanc[1], Stefan Imobersteg[1], Martin Pruschy[3], Paola Picotti[2], Roger Schibli[1, 4], Martin Behe[1]

[#] Correspondence to: Michal Grzmil. Center for Radiopharmaceutical Sciences, Paul Scherrer Institute, Villigen, Switzerland

Tel.: +41 56 310 2857 Fax: +41 56 310 2849

E-mail: michal.grzmil@psi.ch

[1] *Center for Radiopharmaceutical Sciences, Paul Scherrer Institute, Villigen, Switzerland*

[2] *Institute of Molecular Systems Biology, Department of Biology, ETH Zurich, Switzerland*

[3] *Department of Radiation Oncology, University Hospital Zurich, Switzerland*

[4] *Department of Chemistry and Applied Biosciences, ETH Zurich, Switzerland*

## Methods

### Radiolabeling of minigastrin

N-terminal DOTA-conjugated gastrin analogue PP-F11N (DOTA-(DGlu)$_6$-Ala-Tyr-Gly-Trp-Nle-Asp-Phe) was from PSL GmbH, whereas lutetium-177 chloride solution from ITG GmbH. For labeling, 1:30 [$^{177}$Lu]Lu/PP-F11N ratio (24.1 MBq [$^{177}$Lu]Lu per nmol of PP-F11N) was prepared in 0.4 M ammonium acetate buffer (pH 5.5) containing ascorbic acid in a final volume of 50 µL and the labeling was carried out at 90 °C for 15 min. The lutetium-177 incorporation was analyzed by standard HPLC using a C18 column and reached above 95 % efficiency (data not shown). Directly after labeling, the gamma counter was used to prepare appropriate dilutions.

### Cell culture, treatments and proliferation assay

Human epidermoid carcinoma A431 stable cell line that overexpress CCKBR was generated and kindly provided by Dr. Luigi Aloj [1] and cultured in DMEM supplemented with 10% FCS, 2 mM glutamine and antibiotics (0.1 mg/mL streptomycin, 100 IU penicillin) at 37 °C and 5% $CO_2$. Cilengitide (Tocris) was diluted in water and used for the treatment alone or in combination with a radiolabeled minigastrin. Screen-well kinase inhibitor library comprising 80 inhibitors diluted in DMSO (10 mM) was from Enzo Life Sciences AG. For combinatory treatments 2000 cells seeded in 96-well plates were incubated with DMEM containing 0.1% BSA and 1-10 MBq [$^{177}$Lu]Lu-PP-F11N /ml. After 2h, PBS-washed cells were further incubated with 10 µM kinase inhibitors for 24 h and subjected to proliferation assay. External irradiation was performed using an Xstrahl 200 kV X-Ray unit at 100 cGy/minute. The proliferation was analyzed after 24, 48 and 72 h by a CellTiter 96 AQueous Non-Radioactive Cell Proliferation Assay (Promega) according to the manufacturer's instructions. Absorbance of MTS bio-reduced into a formazan was measured at 570 nm with a reference of 650 nm using a MicroPlate Reader (PerkinElmer). The assay was performed in triplicate. One-way ANOVA followed by multiple comparison tests were performed for three or more groups (GraphPad Prism 7.00), whereas two-tailed Student's t tests were performed for analysis of two groups. Values of $P<0.05$ were considered statistically significant.

**Preparation of tryptic peptides and phosphopeptide enrichment**

Cells were grown on 100 mm plates and 100% confluent cultures were subjected to 2 h incubation with Lu-177 labeled PP-F11N (5 MBq per ml of medium) or to external irradiation using (4Gy) as described above. Two hours after internal or external irradiation the total protein lysates were prepared in 8M urea lysis buffer in 0.1 M Ammonium Bicarbonate (Ambic) supplemented with cOmplete mini protease and PhosSTOP phosphatase inhibitors (Roche). Reduction and alkylation steps of each 6 mg protein sample were accomplished by 30 min incubation at 37°C in 12 mM dithiothreitol (DTT) followed by 30 min incubation at 25°C in 40 mM iodoacetamide (IAA) in the dark, respectively. Diluted in 0.1 M Ambic to the final 2M urea concentration proteins were cleaved with trypsin (Promega) at 37°C overnight. The reaction was stopped by adding formic acid to 2% of final concentration (pH < 3). Desalting was accomplished on Sep-Pack-C18 cartridge (Waters Corp.) according to the manufacturer's instructions. For phosphopeptide enrichment 1 mg of $TiO_2$ beads (methanol-activated, and 80% ACN, 6% TFA washed) were incubated at RT for 1 h per 1 mg of protein sample. Beads were washed with 80% ACN, 6% TFA than with 50% ACN, 0.1% TFA, 200 mM NaCl and finally with 50% ACN, 0.1% TFA solution. The elution was performed by using 5% $NH_4$. The eluted phosphopeptide solution was acidified to pH < 3 by adding 100% TFA and subjected to a desalting step using UltraMicroSpin columns (The Nest Group) according to the manufacturer's instructions. The peptides and TiO2-enriched phosphopeptide samples were subjected to mass spectrometry for proteomics and phosphoproteomics analysis, respectively.

**Mass spectrometry**

The samples were measured on an EASY-nLC 1000 (Thermo Fisher) coupled to a Q Exactive Plus mass spectrometer (Thermo Fisher). Peptides were separated on a column (40 cm x 75 μm), packed in-house with reversed-phase ReproSil-Pur C18- AQ resin (1.9 μm, Dr. Maisch). Peptides were eluted for 110 min using a segmented linear gradient of 5% to 40% solvent B (99.9 % acetonitrile, 0.1 % formic acid) at a flow-rate of 300 nL/min. Survey full-scan mass spectra were acquired with mass range 350-1500 m/z, at a resolution of 70.000 at 200 m/z and the 20 most intense ions above an intensity of 3.6e4 were

sequentially isolated, fragmented (normalized collision energy 25 eV) and measured at a resolution of 17.500 at 200 m/z. Peptides with a charge of +1 or with unassigned charge state were excluded from fragmentation for MS2, and a dynamic exclusion of 30 s was applied. Ions were accumulated to a target value of 3e6 for MS1 and of 1e5 for MS2. Obtained raw files were subjected to MaxQuant for label-free quantification (Modifications: oxidation, acetylation (Protein N-term) and additionally phosphorylation (STY) for phosphoproteomics; Max. miss cleaved 2; Min. ratio count 1 or 2 for phosphoproteomics and proteomics groups, respectively). The false discovery rate (FDR) for identification was 0.01. MaxQuant Phospho(STY)Sites.txt and ProteinGroups.txt tables were used for statistical analysis by using Perseus software (http://www.perseus-framework.org/). Reverse hits and potential contaminants were removed from analysis. Imputation of maximum three missing values from normal distribution was allowed for peptides with acquired intensities in all three replicates from one condition (control or treatment). For relative phosphopeptide and protein abundance analysis, calculated log2 transformed ratios between peptide intensities from treatment and corresponding control groups were used for volcano plots and significance analysis with FDR of 0.05. The mass spectrometry proteomics (phosphoproteomics) data have been deposited to the ProteomeXchange Consortium via the PRIDE [2, 3] partner repository with the dataset identifier PXD036034.

**Bioinformatics**

Selected protein lists with significant changes were subjected to STRING version 10 (http://string-db.org/) [4] and the interaction networks were obtained on the evidence basis with active interaction sources; experiments and databases (high confidence 0.7). For the network visualization k-means clustering was used and the disconnected nodes were disabled. For pathway (BioCarta, KEGG pathways) or biological process (GO term) analysis DAVID web-based tool [5] was used. *P*-values and the fold enrichments were obtained for each identified term.

**WB analysis**

Antibodies against phospho-P53 at S15 (16G8), phospho-ERK1/2 at T202/Y204 (D13.14.4E), phospho-c-JUN at S73 (9164), CYR61 (D4H5D), P53 (7F5), ERK1/2 (9102) and GAPDH (14C10) were obtained from Cell Signaling Technology. Cells were homogenized in lysis buffer (50 mM Tris-HCl pH 7.5, 150 mM NaCl, 1 % Triton X, 0.1 % SDS supplemented with 1 mM sodium orthovanadate, 1 mM NaF and protease inhibitor cocktail (Roche)). Aliquots of 50 μg protein extracts were separated by SDS-PAGE and transferred to PVDF membranes (Millipore) by electroblotting. Membranes were blocked with 5 % skim milk in TBST (0.1% Tween 20) for 1 h, and incubated with 2 % BSA in TBST overnight with the primary antibody followed by 2 h incubation with HRP-conjugated secondary antibody. Protein-specific signals were detected by a chemiluminescence reagent (ECL) and signals were acquired by using ImageQuant RT ECL Imager (GE Healthcare).

**Animal study**

CD-1 female nude mice (Charles Rivers, Germany) were subcutaneously injected with 5 x $10^6$ of A431/CCKBR cells in 0.1 mL of phosphate-buffered saline (PBS) containing 0.9 % NaCl. Eight days after tumor implantation, animals with tumors (≥ 0.15 $cm^3$) were randomly distributed into experimental groups. Erlotinib (10 mg/kg), Cilengentide (12.5 mg/kg) and PBS (control) were administered via intraperitoneal injection daily. On the second of treatment, HPLC-purified 60 MBq of [$^{177}$Lu]Lu-PPF11N (721 MBq/nmol) in 100 μL PBS was injected intravenously. The control group was injected with 100 μL PBS. The tumor diameters were measured non-invasively with a caliper and the volume was calculated by using the formula $V = (W^2 \times L)/2$. The nude mice were sacrificed when the tumor volume exceeded 1.5 $cm^3$. The mice with ulcerated tumors, present in all groups, were sacrificed prematurely and were excluded from the analysis. All experiments were performed in accordance with Swiss Animal Protection Laws. GraphPad Prism 7.00 for Windows was used for statistical analysis including one-way ANOVA followed by multiple comparisons for tumor growth analysis and Long-rank (Mantel-Cox) test for survival curve comparison.

**References for Supplementary Methods**


1. Aloj L, Caraco C, Panico M, Zannetti A, Del Vecchio S, Tesauro D, et al. In vitro and in vivo evaluation of 111In-DTPAGlu-G-CCK8 for cholecystokinin-B receptor imaging. J Nucl Med. 2004; 45: 485-94.
2. Perez-Riverol Y, Bai J, Bandla C, Hewapathirana S, García-Seisdedos D, Kamatchinathan S, et al. The PRIDE database resources in 2022: a hub for mass spectrometry-based proteomics evidences. Nucleic Acids Res. 2022; 50:D543-D552.
3. Deutsch EW, Bandeira N, Sharma V, Perez-Riverol Y, Carver JJ, Kundu DJ, et al. The ProteomeXchange consortium in 2020: enabling 'big data' approaches in proteomics. Nucleic Acids Res. 2020; 48:D1145-D1152.
4. Szklarczyk D, Franceschini A, Wyder S, Forslund K, Heller D, Huerta-Cepas J, et al. STRING v10: protein-protein interaction networks, integrated over the tree of life. Nucleic Acids Res. 2015; 43: D447-52.
5. Dennis G, Jr., Sherman BT, Hosack DA, Yang J, Gao W, Lane HC, et al. DAVID: Database for Annotation, Visualization, and Integrated Discovery. Genome Biol. 2003; 4: P3.
